# Cross-Border Access to Clinical Trials in the EU: Exploratory Study on Needs and Reality

**DOI:** 10.3389/fmed.2020.585722

**Published:** 2020-10-22

**Authors:** Teodora Lalova, Cristina Padeanu, Anastassia Negrouk, Denis Lacombe, Jan Geissler, Ingrid Klingmann, Isabelle Huys

**Affiliations:** ^1^Department of Pharmaceutical and Pharmacological Sciences, Clinical Pharmacology and Pharmacotherapy, KU Leuven, Leuven, Belgium; ^2^Center for IT & IP law (CiTiP), KU Leuven, Leuven, Belgium; ^3^European Forum for Good Clinical Practice, Brussels, Belgium; ^4^European Organization for Research and Treatment of Cancer, Brussels, Belgium; ^5^Patvocates, Munich, Germany

**Keywords:** clinical trials, patient rights, cross-border healthcare, cross-border access, exploratory study, clinical trials sponsors, pharmaceutical industry

## Abstract

**Objectives:** To analyze the current situation of cross-border access to clinical trials in the EU with an overview of stakeholders' real-life experience, and to identify the needs, challenges, and potential for facilitation of cross-border access.

**Methods:** We employed a mixed methods design. Semi-structured interviews and an online survey were conducted with a wide range of stakeholders: patient representatives, investigators/physicians, policy and regulatory experts, academic and commercial sponsor representatives, ethics committee members. Interviews underwent a framework analysis. The survey was analyzed descriptively.

**Results:** Three hundred ninety six individuals responded to the survey. The majority were investigators/physicians (46%) and patient representatives (33%). Thirty eight individuals were interviewed. The majority were investigators/physicians (29%) and patient representatives (29%). All European regions were represented in the study. The highest response rate was received from residents of Western European countries (38% of survey respondents, 45% of interviewees), the lowest from Eastern Europe (9% of survey respondents, 5% of interviewees). The study suggested that cross-border participation in clinical trials occurs in practice, however very rarely. Ninety two percentage of survey respondents and the majority of interviewees perceived as needed the possibility to access clinical trials abroad. However, most interviewees also opined that patients ideally should not have to travel in order to access experimental treatment. The lack of access to treatment in the home country of the patient was described as the main motivation to participate in a clinical trial in another country. The logistical and financial burden for patients was perceived as the biggest challenge. Different stakeholders expressed diverging opinions regarding the allocation of financial and organizational responsibility for enabling cross-border access to clinical trials. Participants provided a number of proposals for improving the current system, which were carefully evaluated by the research team and informed future recommendations.

**Conclusions:** Participation in clinical trials abroad is happening rarely but should be facilitated. There was a consensus on the need for reliable and accessible information regarding practical aspects, as well as multi-stakeholder, multi-national recommendations on existing options and best practice on cross-border access to clinical trials. Broader interdisciplinary research is recommended before discussing options in the EU legislative framework to enable clearly defined conditions for cross-border access to clinical trials.

## Introduction

Clinical trials investigating new therapy concepts are of high interest to patients with severe and life-threatening conditions such as cancer (1–4) and rare diseases. In the European Union, the opportunities for joining a clinical trial differ per country. Over the past 10 years, phase 1 clinical trials and phase 3 trials for rare diseases remain concentrated in Western European countries (see Supplementary Material 1). On average, Eastern Europe has seen one-third fewer such trials than Western Europe, for example. For the same period, phase III clinical trials (all indications) were distributed relatively equally, which is in line with previously reported trends about the increasing clinical trial activity of pharmaceutical companies in Western and Eastern Europe (5, 6). Numerous regulatory and legal barriers make the conduct of pan-European clinical trials challenging for sponsors (7, 8) and many different criteria inform decision-making for site selection (9–11). In recent years, new molecularly driven trial designs impose hurdles linked to the prevalence of biomarkers and potentially severe limitations in the availability of a specific study population (12).

Based on the EU's principle on freedom of movement^1^, participation in a clinical trial abroad is theoretically possible. While there is no specific European legislation on facilitation of cross-border clinical trial participation, frameworks exist, notably the Nordic Network for Early Cancer Trials (Nordic NECT) (13). The Nordic NECT is designed to promote patient access to new investigational drugs and access to phase I and early phase II programs in Norway, Denmark, Finland, and Sweden, and among its objectives *is “work for a bilateral agreement between the Nordic countries allowing for inclusion of patients in early clinical trial protocols across the borders.”* How to access a study abroad is also a discussion and support topic for patient organizations (14, 15) and research charities (16). However, to the best of our knowledge, the issue has not been systematically investigated.

Participation in clinical trials in another EU Member State or outside the EU is not included in the scope of Directive 2011/24/EU on the application of patients' rights in cross-border healthcare (hereafter the “Directive”). The Directive sets out the conditions under which patients may travel to another EU country to receive medical care and can claim reimbursement. It was the result of a difficult compromise between member states and codifies a line of jurisprudence developed by the Court of Justice of the EU (CJEU) of which clinical research was not part (17). Further, the Directive complements the existing social security regulations (18). Pursuant to Article 7(1) of the Directive, the Member State of affiliation (patient's home country) shall ensure that the costs incurred by an insured person who receives cross-border healthcare are reimbursed, if the healthcare in question is among the benefits to which the insured person is entitled in said Member State. Article 7(4) further specifies that the costs of cross-border healthcare shall be reimbursed or paid directly by the home country to the amount of costs that would have been assumed by it, had this healthcare been provided in its territory.

In addition to the reimbursement mechanism, the Directive has several key features that are of interest for the topic of cross-border access to clinical trials:

First, it established the creation of National Contact Points (NCPs) in each Member States. Their objective is to provide patients with information concerning, *inter alia*, healthcare providers, patients' rights, and complaints procedures.

Second, it establishes the launch of the European Reference Networks (ERNs)^2^ which marked a major change for the delivery of quality and accessible cross-border healthcare to EU citizens. The objectives of the ERNs^3^ designate them as privileged hubs for research. At the current moment, there are 24 ERNs which work in a range of diseases across 26 countries, with cancer-related rare diseases covered by ERN-EuroBloodNET, ERN-Genturis, ERN-PaedCan, and ERN-EURACAN. However, their long-term sustainability is challenged by limited funding, lack of awareness among patients and healthcare professionals, lack of support from hospital managers, and the limited human resources available to work on ERN-related initiatives (19). Encouraged by the European Federation of Pharmaceutical Industry Associations (EFPIA)'s Oncology Platform, and supported with an unrestricted grant, the European Forum for Good Clinical Practice (EFGCP), the European Organization for Research and Treatment of Cancer (EORTC), KU Leuven, and Patvocates formed a research consortium that set out to investigate the current state of cross-border access to clinical trials in the EU and to systematically research the needs, challenges, and potential for facilitation in the view of concerned stakeholders.

## Methods

### General Design

We employed a mixed methods approach in form of a triangulation design, in which qualitative and quantitative methods were applied during the same timeframe and with equal weight (20). A convergence model was used. The results from the qualitative and quantitative arms of the study were collected and analyzed separately, and then compared during interpretation (20). An online survey and semi-structured interviews were performed, targeting key stakeholders involved in the conduct of EU clinical trials. The two questionnaires explored similar topics, namely the current situation concerning cross-border access to clinical trials and the potential for facilitation in the field. The survey was active between May 29 and July 31, 2019. The interviews were conducted between May and September 2019.

### Participants

The survey was broadly distributed to individuals via the pan-European networks of the research consortium, and via social media (e.g., LinkedIn, Twitter). The survey targeted six stakeholder groups: (1) investigators/physicians (A), (2) representatives of patient organizations (B1), (3) individual patients/carers (B2), (4) commercial and academic sponsors of clinical trials (C+F), (5) ethics committees (E), and (6) regulators (H). In addition, it provided an “Other” option for individuals belonging to other stakeholder groups (I).

Participants for the interviews were recruited via a combination of purposive and snowball^4^ sampling approach (see Table 1). In total, 86 individuals were invited. Thirty-eight semi-structured interviews were conducted with (1) investigators/physicians (A), (2) patient representatives (B), (3) pharmaceutical industry representatives (C), (4) policy experts (D), (5) ethics committee representatives (E), (6) academic sponsors of clinical trials (F), and (7) national contact points (NCPs) (G). The aim was to achieve an even distribution of representatives across stakeholder groups. The research project strived for a balanced representation of the EU countries.

**Table 1:** Inclusion criteria for the recruitment of representatives of each stakeholder group included in the interviews.

**Stakeholder group**	**Inclusion criteria**
Investigators/Physicians	•Has been actively involved in clinical trials as a principal investigator or has experience with referring patients abroad/receiving foreign patients •Holds a senior position at a University hospital •Speaks fluent English •Works or has worked in a Member State of the European Union
Patient representatives	•Has experience working as a patient representative •Speaks fluent English •Works or has worked in a Member State of the European Union
Pharmaceutical industry representatives	•Holds a senior management position at a pharmaceutical company •Is actively involved in decision-making •Speaks fluent English
Policy experts	•Has professional experience with clinical research and patient recruitment •Speaks fluent English •Works or has worked in a Member State of the European Union
Ethics committees representatives	•Is member of an ethics committee based in a Member State of the European Union •Speaks fluent English •Works or has worked in a Member State of the European Union
Academic clinical trials sponsors	•Holds a senior position at an organization involved in the conduct of academic clinical trials •Speaks fluent English •Works or has worked in a Member State of the European Union
National contact points	•Holds a position at a designated National contact point as established under Directive 2011/24/EU on patients' rights in cross-border healthcare •Speaks fluent English •Works or has worked in a Member State of the European Union

### Conduct

The research team designed a survey questionnaire consisting of 25 questions (Supplementary Material 2). The choice of possible answers was proposed by the experienced patient representatives and healthcare professionals who collaborated in the study design, and was also based on information collected through literature review. The questionnaire was in English and was uploaded on the SurveyMonkey® online software. In order to participate, respondents had to give informed consent.

For the interviews, an interview guide was developed based on a literature review and input from co-authors with practical experience in the field. The guide consisted of 23 open-ended questions (Supplementary Material 3) and was piloted with three persons who were not part of the study but had relevant professional backgrounds (medical oncologist, pediatric oncologist, and a chair of an ethics committee). Interviews were conducted via Skype® or in person by three members of the research team. Each interviewee signed an informed consent form prior to the interview. The sessions were digitally recorded and all interviews were subsequently transcribed *ad verbatim* by a third party.

### Analysis

Survey data was analyzed anonymously, using descriptive statistics on version 9.4 of the SAS System for Windows. The figures in this paper were generated in Excel. Percentages were calculated based on the number of respondents for each specific question (Supplementary Material 4). The interviews were analyzed according to the framework method (22), using the NVivo® software. The Qualitative Analysis Guide of Leuven (23) was followed. The coding of all transcripts was performed by one researcher, based on a working analytical framework. The framework was prepared in three stages: (1) two members of the research team coded individually five transcripts, (2) the codes were compared, and (3) the ambiguities and overlaps were cleared out with the guidance of the author most experienced in this methodology.

## Results

Survey results are presented first, followed by the in-depth interview insights. The presentation is structured according to the main themes explored in the study. Reference is made to survey questions' (Q) numbers. Quotes from the interviews are followed by a reference to the stakeholder group to which the interviewee belonged (see Methods Participants).

### Demographics

**Figure 1:**
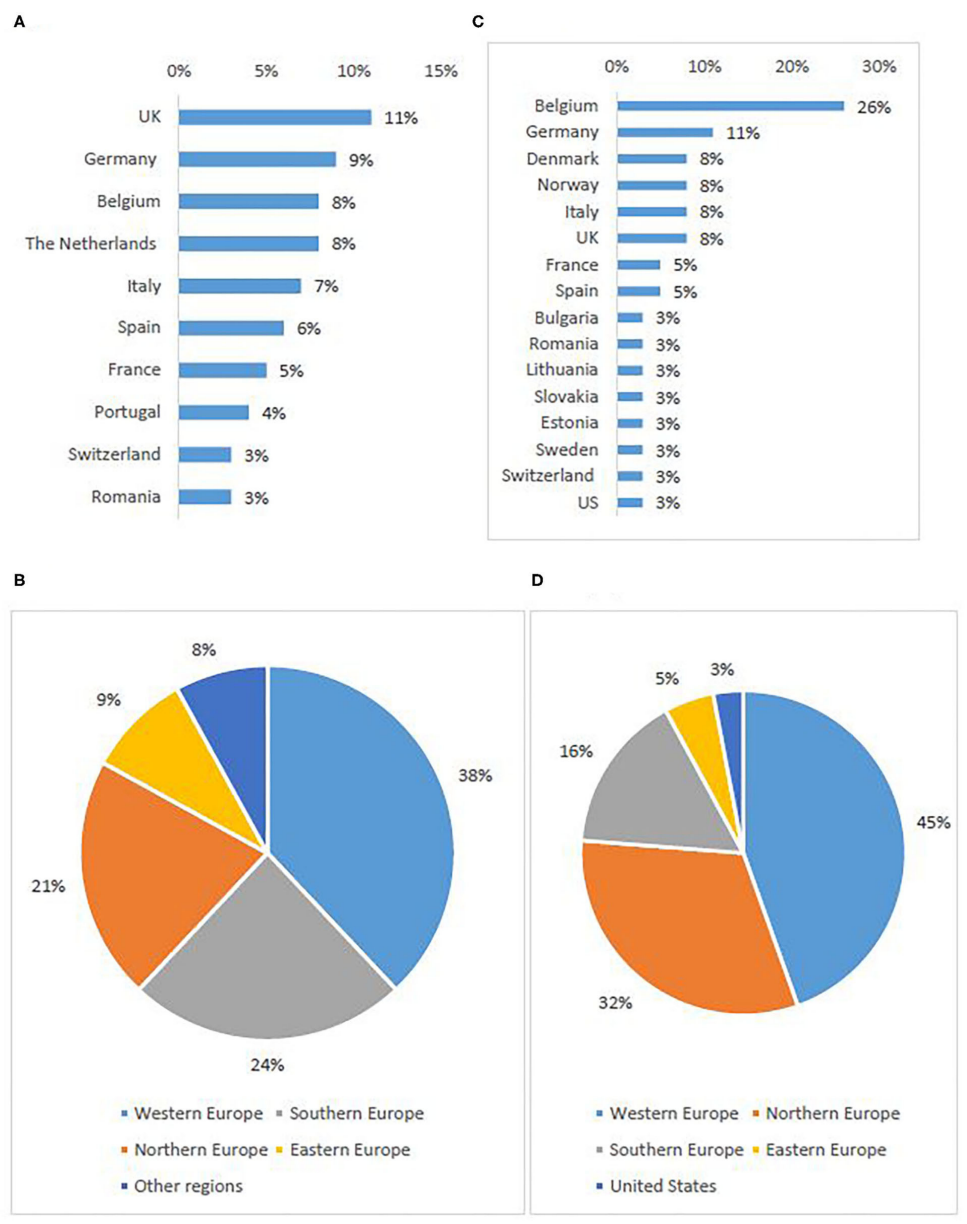
Stakeholder representation: survey respondents per first ten represented countries **(A)** and per sub-region **(B)**, interviewees per country **(C)** and per sub-region^5^ **(D)**.

In total, 396 individuals participated in the survey, most of which were investigators/physicians (46%) and patient representatives (33%) (Table 2A). The highest response rate was received from Western European countries (38%), the lowest from Eastern Europe (9%) (Figures 1A,B).

**Table 2:** Stakeholder groups ascribed codes and representation (No. and %) in the survey **(A)** and interviews **(B)**.

**Stakeholder group (survey)**	**Code of the stakeholder group**	**No of Survey respondents**	**% of the total number of survey respondents**
**(A) SURVEY**			
Investigators/physicians	A	183	46
Patient representatives	B		B (B1+B2) comprise 33
	B1 (representatives of patient organizations)	91	23
	B2 (individual patients/carers)	40	10
Sponsors of clinical trials (commercial or academic)	C+F	38	10
Ethics committees	E	4	1
Regulators	H	4	1
Other	I	36	9
Total	396		
**Stakeholder group**	**Code of the stakeholder group**	**No of Interviewees**	**% of the total number of interviewees**
**(B) INTERVIEWS**			
Investigators/Physicians	A	11	29
Patient representatives	B	11	29
Pharmaceutical industry representatives	C	5	13
Policy experts	D	6	16
Ethics committees representatives	E	3	8
Academic clinical trials sponsors	F	1	3
National contact points	G	1	3
Total	38		

Out of 86 invited experts, 38 agreed to be interviewed. Investigators/physicians and patient representatives demonstrated the highest interest to participate (Table 2B). One NCP representative agreed to contribute. The others did not respond to the invitation or refused to participate, citing a lack of experience with the topic. The majority of investigators involved in the study had expertise in oncology. Although the views of pediatric experts were included, the study focused primarily on participation of adult subjects in clinical trials. The highest participation rate was from Western European countries (45%), the lowest from Eastern Europe (5%) (Figures 1C,D).

### Occurrence of Cross-Border Access to Clinical Trials

As there are no official statistics, several of the questions referred to the extent to which patients currently participate in clinical trials outside of their home countries.

#### Experience With Cross-Border Access to Clinical Trials

Forty-four percent of the survey respondents (Q4) had no experience with cross-border access to clinical trials. Only 4% reported having participated in a cross-border clinical trial as patients themselves. The rest of the responses were distributed equally between different types of involvement, such as advising patients (23%), enrolling patients (22%), or designing cross-border clinical trials (22%). In addition, the majority of survey participants (Q5) indicated that clinical trial protocols neither forbid, nor foresee cross-border participation (48%, 69%).

The majority of interviewees described their knowledge in cross-border access to clinical trials as limited. However, direct experience was reported by more than half of participants (A, B, C, D, E), and similarly to the survey consisted of referring patients to clinical trials abroad, enrolling foreign patients, or assisting patients in finding a relevant trial abroad. The rest of the participants (B, C, D, E, F, G), indicated indirect experience gained through discussions with experts with direct experience (e.g., investigators), or through any other means (e.g., reports, media articles, participation in events). All interviewees from the investigators/physicians stakeholder group had direct experience, whereas indirect experience was more prevalent within the groups of patient representatives, policy experts, and the pharmaceutical industry representatives. In addition, seven interviewees (B, D, F) had gathered experience in personal capacity, as patient, carer, or volunteer.

#### Frequency, Increase, or Decrease in Cross-Border Access to Clinical Trials

**Figure 2:**
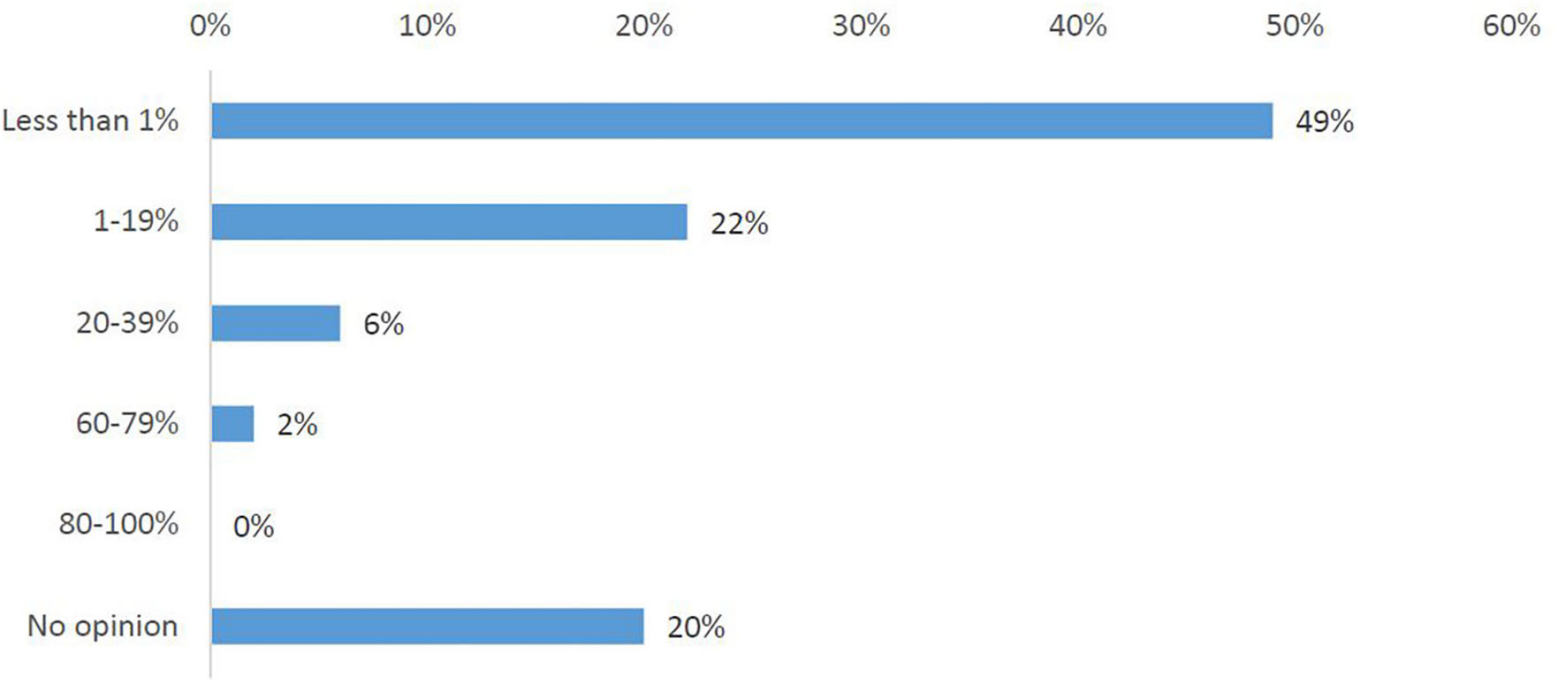
Investigators' responses to the survey question, “What has been the highest percentage of foreign patients you have had in any of your clinical trials?”.

In total, 75% of the survey respondents stated that cross-border participation in clinical trials occurs rarely (Q9). One-third (34%) of respondents did observe an increase in requests for participation in clinical trials abroad, one-third (33%) did not see this trend and one-third (33%) had no opinion (Q6A). Nearly half of the participants (43%) had, in fact, not observed any increase in the inclusion of foreign patients into a trial, with 34% stating they do not have information about this (Q6B). Half of the investigators (Q7) responded that they have observed <1% foreign patients participating in any of their clinical trials (Figure 2).

The survey results were corroborated in the interviews. Although many interviewees refused to comment, some were of the opinion that patients rarely express interest in joining clinical trials abroad. Others stated, with various degrees of certainty, that cross-border participation in clinical trials occurs in the EU, although in a limited manner, and that currently there is an increased interest in it. Several potential reasons for this increase were listed, namely higher patient awareness of clinical trials, advances in medicine that are not yet broadly available (e.g., new technologies only available in specialized centers abroad), and the EU freedom of movement.

“*Once somebody realizes that there is a freedom of movement, there's very little that can keep them back. (…) you cannot put the spirit back into the bottle. People will move, and especially if their life and if their health depends on it.” (B)*

#### Motivations for Cross-Border Access to Clinical Trials

##### Motivations for patients to participate

**Figure 3:**
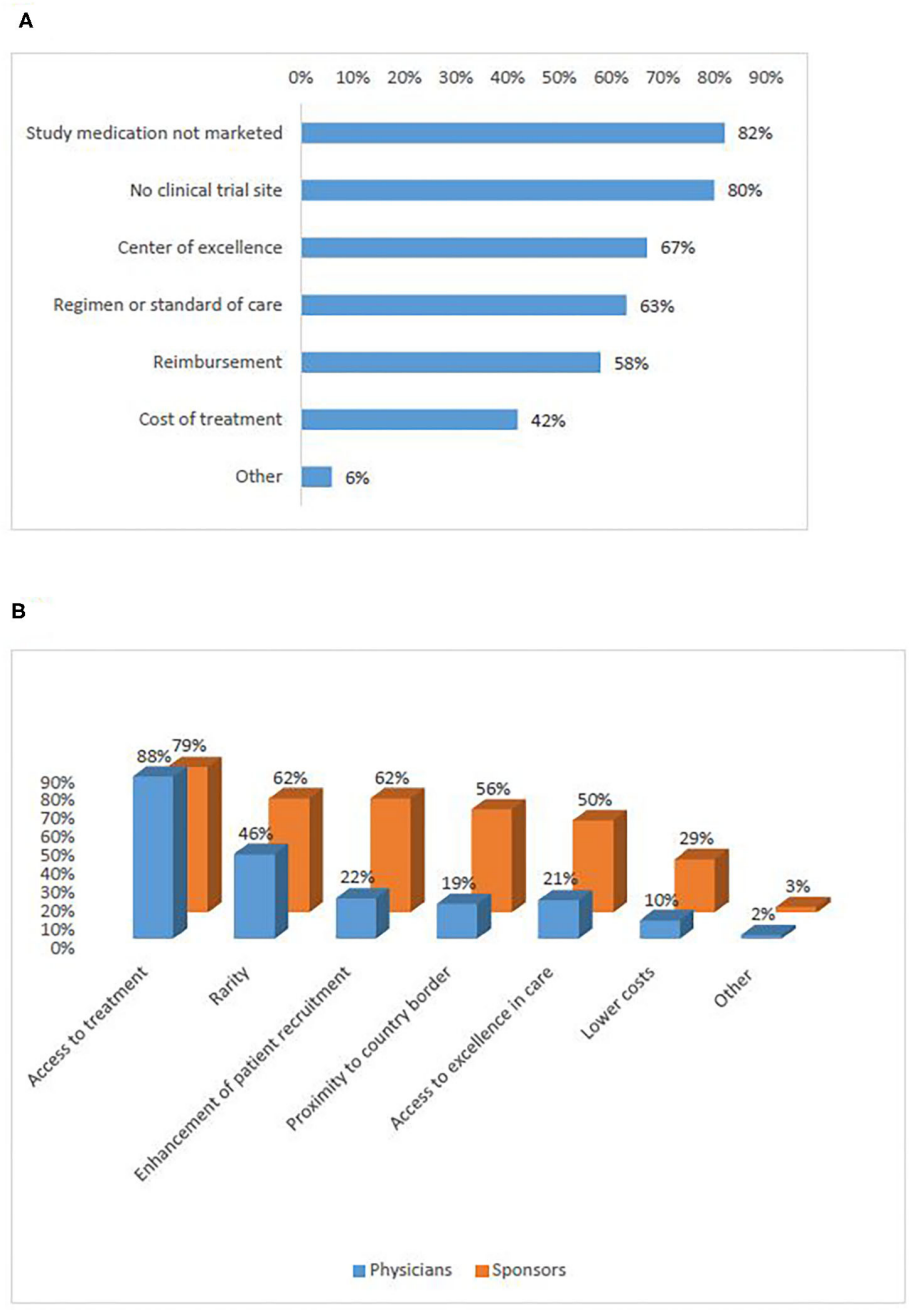
Motivations for cross-border access to clinical trials (survey): of patients **(A)**, sponsors and physicians **(B)**.

According to patient representatives who participated in the survey, the strongest reason to seek participation in a trial abroad was access to a new treatment that is either not marketed (82%), or not available in a similar trial in their country of residence (80%) (Figure 3A) (Q11). Interviewees also identified access to treatment as the main reason to seek or recommend participation in a clinical trial abroad. However, the interviews showed that it is difficult to find a common understanding of the notion “access to treatment.” The majority of participants elaborated that the concept refers to a new promising treatment that is not available in the patient's home country mainly in the following four situations: (1) in the case of rare diseases with no existing treatment; (2) when all available lines of therapy have been exhausted; (3) when the treatment is not reimbursed by the healthcare system in the patient's home country; (4) when the study uses specific technology that is not yet available in the home country of the patient (e.g., genome sequencing, proton beam therapy). Other reasons to seek participation abroad are linked to physicians' advice or willingness to contribute to science. Additionally, interviewees mentioned reasons to seek participation in a trial abroad even when a site of the same trial is open in the patient's home country. These included living geographically closer to the site open abroad or having a higher trust in the foreign country's healthcare system or the foreign center where the clinical trials is conducted.

“*You might even get a trial in your own country (…) but you know that the center that is running this is by far not as good as a specialized center for that rare disease in another country, so you might want to go to [names of hospitals edited out for confidentiality reasons] because you know they are specialized in that specific cancer and there's no one better than their expert” (B)*

Interviewees elaborated as well on the reasons not to participate in a clinical trial abroad. The first was the reluctance to travel, due to either (a) the vulnerable state of the patient; (b) the high trust in the home country's healthcare system; or (c) the fact that most clinical trials are available in the patient's home country (as stated by interviewees based in Western Europe). The second was the idea that the general view on clinical trials in some EU countries may be more critical and/or distrustful than in others, which might affect the willingness of individual patients to participate in a study conducted abroad. Finally, the risk of being randomized in the placebo arm of a clinical trial can be decisive. In the words of one participants (B)*: “I would try do to everything to avoid being randomized into a placebo arm, because that would be unacceptable risk.”* However, the interviewee did not regard placebo-controlled trials as a block to going abroad, as long as there was an option to get switched to the active arm when medically required.

##### Motivations for physicians to recommend participation

In the survey, the reasons for which physicians would recommend participation in a clinical trial abroad were distributed similarly to the motivations of sponsors to recruit foreign patients (Figure 3B) (Q12, Q13). Both groups ranked access to treatment highest (88%, 79%). Rarity (i.e., when the incidence of patients with the protocol-required very specific in- and exclusion criteria is low) and enhancement of recruitment were also frequently selected. In the interviews, investigators/physicians and sponsors added the situations when it is unfeasible to open investigational sites in many EU Member States due to the heavy regulatory and legal burden of doing so. Recruitment enhancement was seen as particularly relevant in the context of precision medicine when very specific inclusion criteria would be present, and also in rare diseases.

“*In rare diseases you have no choice” (C)*.

One representative (C) stressed, however, that the majority of patients that participate in clinical trials are recruited locally. Reasons for this include the fact that sponsors would seek to enable participation in the nearest hospital possible. Furthermore, clinical trials subjects have to comply with strict and frequent visits to the investigational site, and this was considered burdensome for foreign patients.

### European Countries Attractive for Patients to Seek Participation in a Clinical Trial

**Figure 4:**
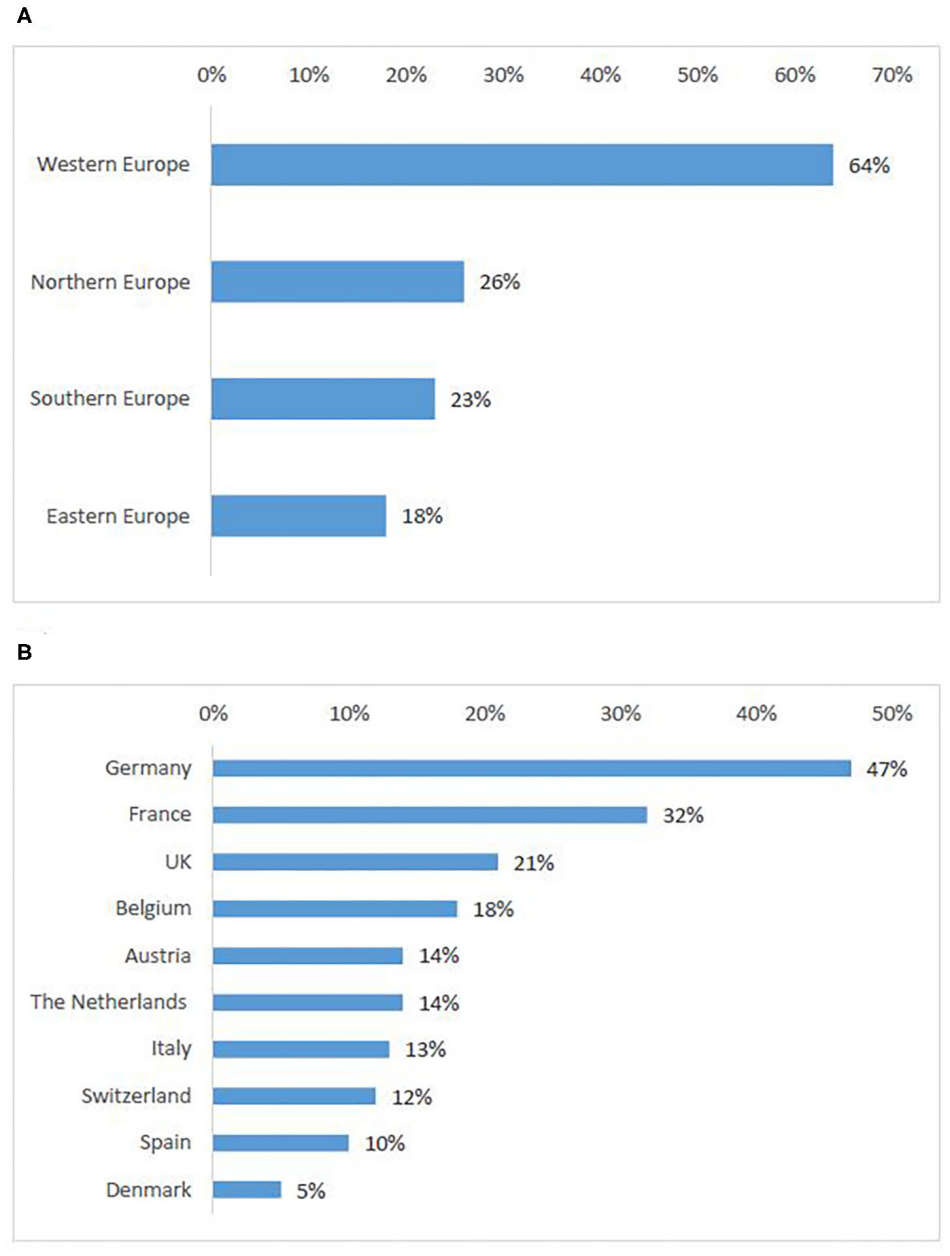
European countries attractive for patients to seek participation in clinical trials: ranking per sub-region **(A)**, ten countries most frequently selected by survey participants **(B)**.

Based on the preference shown by survey respondents, Western countries were ranked highest (64%) (Figure 4A) (Q14). Interviewees also cited most frequently countries that were ranked in the top ten produced in the survey (Figure 4B), namely Belgium, Germany, The Netherlands, France, UK, and Spain. Interviewees explained that the sponsors' reasons for choice of these countries were often availability of experienced sites and time to trial approval while the patients' choices were more closely linked to patients' motivations to seek participation in a clinical trial abroad (see Motivations for cross-border access to clinical trials) and countries with the highest number of ongoing trials. Another relevant aspect was mentioned in an interview with a patient representative:

“*It's not about the country, it's where the clinical trials happen. And the clinical trials happen where the markets are. Companies, they frequently prefer to go to the market where they are planning to get reimbursement and then sell their products afterwards.”*

In addition, countries might be attractive for patients in case they are (1) a neighboring country, (2) a country where the language barrier is alleviated (e.g., Belgium with respect to patients coming from France or the Netherlands); (3) trust is present in the excellence of the healthcare system or in the science in that country; (4) culturally similar and can use established frameworks of collaboration (especially prominent in the Nordic region where such frameworks were reported as established with government support); (5) recommended by the treating physician.

### European Countries of Origin of Patients Seeking Participation in a Clinical Trial Conducted Abroad

**Figure 5:**
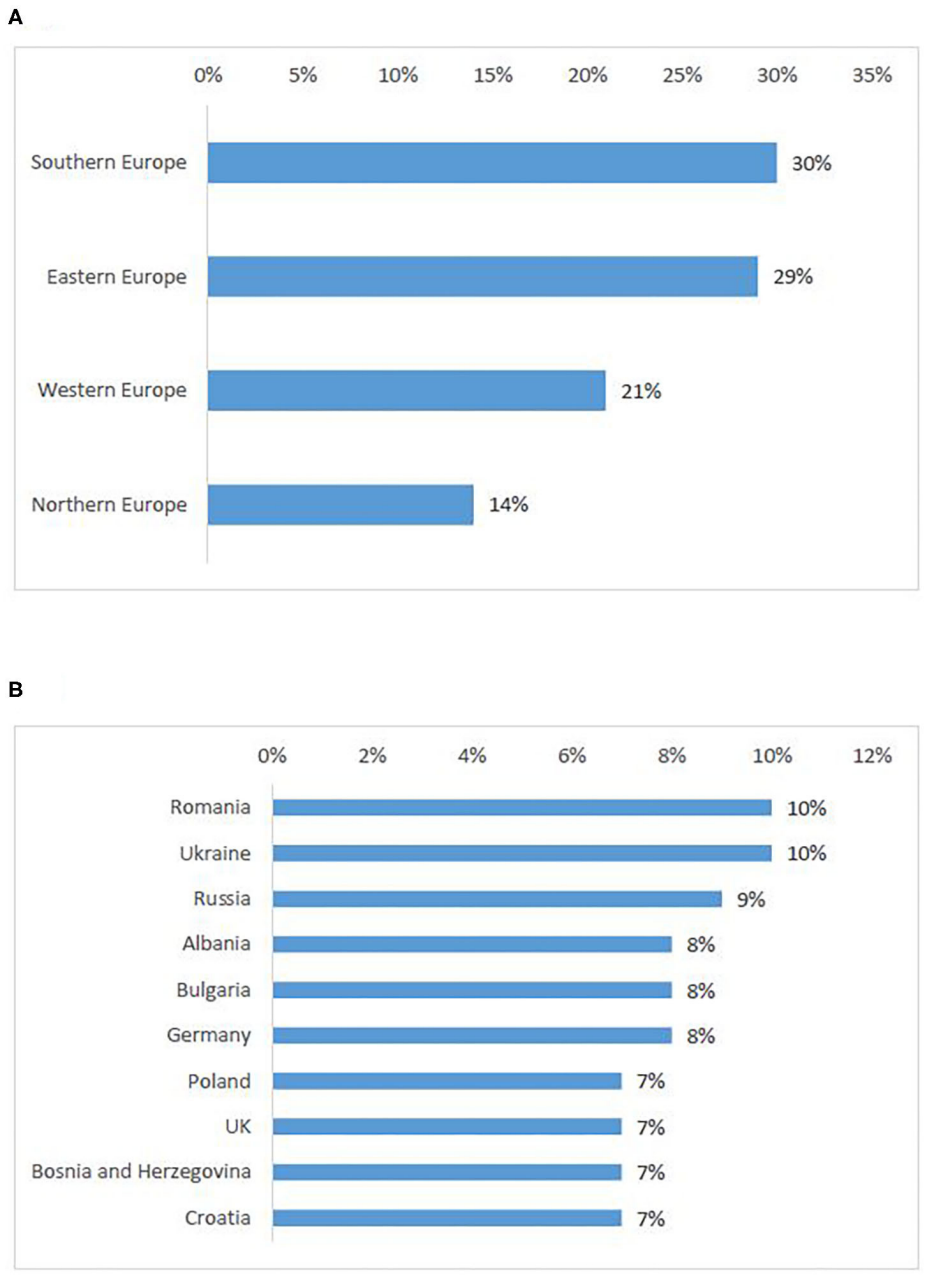
European countries of origin for patients seeking participation in a clinical trial abroad: ranking per sub-region **(A)**, ten countries most frequently selected by survey participants **(B)**.

According to survey respondents, the need for cross-border access to clinical trials is similarly distributed in Southern (30%) and Eastern European countries (29%), whereas Northern Europeans seek the least access to clinical trials abroad (Figure 5A). Romania was the highest-ranking country. Countries that were cited as attractive for cross-border participation (Germany, UK), also appeared in the first ten countries of origin of patients seeking participation abroad (Figure 5B).

The majority of interviewees agreed that the need for cross-border access to clinical trials is “*widely distributed across Europe”* (A), in line with the survey results.

The reasons for patients from certain countries to seek cross-border access to clinical trials mirror the reasons for other countries' attractivity, especially concerning neighbor country location of the site and perceived inferiority of the healthcare system in the own country. However, one Interviewee (A) mentioned that patients who come from countries with less-developed healthcare systems would not primarily seek to enroll in a clinical trial, but would rather seek treatment in the “*private environment”* and as part of the standard clinical practice. Countries with the least open clinical trials include CEE countries (e.g., Bulgaria, Romania) and Greece. However, in situations where no promising new treatment was currently available, patients seemed to look for participation abroad regardless of their geographical location.

### Challenges to Participate in Cross-Border Clinical Trials

**Figure 6:**
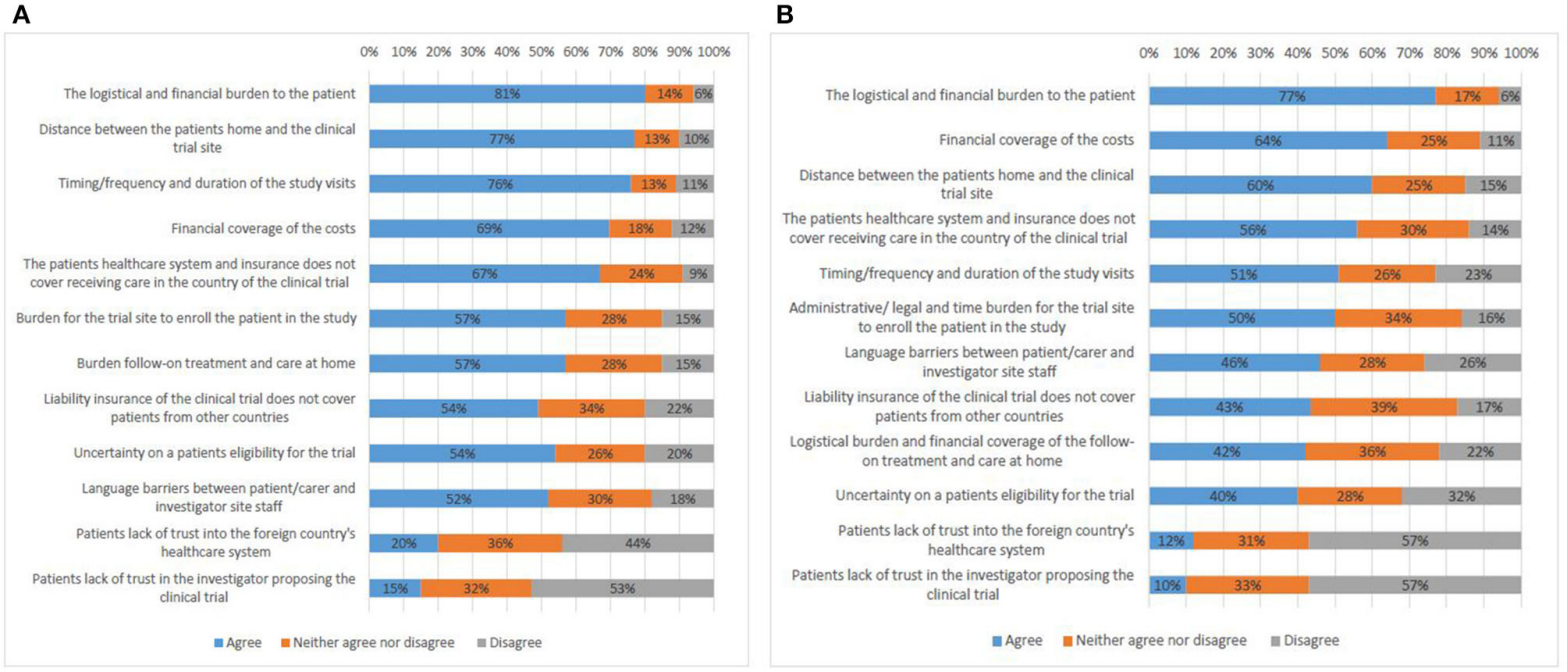
Factors that present a challenge to cross-border access to clinical trials according to survey respondents who do not have experience **(A)**, and survey respondents who have experience **(B)**.

The survey compared the opinions on challenges in cross-border participation in clinical trials from respondents who had not tried to access a clinical trial abroad (Q17) with those from participants with experience (Q18). Both types of respondents answered in a similar way (Figures 6A,B). The logistical and financial burden to the patient was ranked first (81%, 77%).

Interviewees from all stakeholder groups also defined the coverage of costs as the main challenge and made a distinction between two types of costs. On the one hand, there were costs associated with joining the clinical trial. These could include, e.g., travel expenses, accommodation, translation services, and care (hiring a carer or having a family member accompany the patient), or the costs of the baseline therapy in cases where the experimental treatment was an add-on.

“*Not always they have reimbursement of all the clinical procedures in those clinical trials” (A)*

On the other hand, there were costs associated with loss of income, e.g., if the patient and/or the patient's carer had to leave their employment in order to be able to travel abroad.

The survey results positioned the language barrier between patients and investigator site staff relatively low in the overall ranking. On the contrary, all interviewees put a high emphasis on language issues, not only when it comes to communicating with hospital staff, but also with respect to day-to-day life in the host country.

“*Many patients only speak their mother tongue and no other languages.” (A)*

Moreover, language presents a challenge with respect to the understanding and acceptance of the informed consent for participation in a trial. It emerged that some EU Member States have stricter rules than others. For instance, in Germany, “*if you can't read the informed consent, you can't go on a trial. We have been negotiating with them to at least include an English informed consent (…) because that would open it up to many more [patients]”* (B).

Survey participants (both with and without experience) predominantly saw travel distance as a challenge (77%, 60%). In the interviews, representatives of all stakeholder groups indicated travel distance among the main challenges as well. Neighboring countries see higher numbers of patients seeking to join a study across borders.

“*(…) if you have six-month life expectance, I don't think you should use most of the time on traveling around for participation in clinical trial, unless it's a high probability for a long lasting response” (A)*

Issues with patient follow-on treatment at home were ranked highly by the survey participants (57%, 42%). Also most interviewees brought up this challenge. First, lack of clarity existed on how best to allocate responsibility for follow-up care. It is possible that the home country does not possess an equal level of scientific expertise and/or specialized equipment required for the follow-up treatment as the country where the trial was performed. Second, the generation of follow-up data by the home country for a clinical trial abroad raised the question whether every hospital performing this data generation needs to be considered as a clinical trial site with related need for study approval by a competent authority and ethics committee. Finally, participants reported that the home country's health insurance system may refuse to fund follow-up care services for patients who had access to experimental treatment abroad.

Uncertainty on a patient's eligibility for trial participation (54%, 40%) was seen as an important challenge by all interviewees as it is difficult for patients to assess whether or not they might meet the strict eligibility criteria. Furthermore, in case the eligibility assessment involves performing diagnostic tests, the patient may have to travel to the trial site, which would put financial and logistical burden on the patient prior to joining a clinical trial.

A lack of well-structured and easily accessible information about the availability of clinical trials was mentioned as another challenge. Participants described three key aspects that require better organization. First, information should be available about ongoing clinical trials, eligibility criteria, and location of clinical trial sites, as well as an appropriate system for patient referral and for assessing whether a clinical trial is the best option in a given case. Interviewees reported that most patients are not aware of the EU clinical trials register and clinicaltrials.gov database, or do not find these sources user-friendly, hence are seeking information primarily from treating physicians and patient organization/advocacy groups. However, it is possible that physicians are not aware of suitable clinical trials either, or lack motivation to refer patients. Moreover, patient organizations are not always best equipped to disseminate such information due to lack of time and financial resources. Furthermore, sponsor representatives (C, F) shared that patients regularly attempt to contact them directly, seeking possibilities to join a trial abroad. However, pursuant to ICH GCP standards and applicable legislation, the sponsor is not allowed to know the identity of participants in his clinical trials.

Secondly, trustworthy and easily available information for patients about the value of clinical trials in general is lacking as patients might need to be better familiarized with what clinical trials involve and how they could be of benefit. “*Certain patient populations don't even understand what a clinical trial is. So, I think, the first thing is to explain to patients that a clinical trial is not about being a guinea pig, which still, unfortunately, is the kind of popular opinion in many cases”* (B).

Thirdly, information should be available for all involved stakeholders about best practices when joining a clinical trial abroad, e.g., legislation and regulation that must be taken into account.

Another obstacle discussed by all interviewees related to the legal and regulatory requirements. It was perceived challenging to comply with the EU data protection legal framework, both when assessing eligibility of patients residing abroad, and for conducting pan-European clinical trials. In addition, the lack of a legal framework and regulatory infrastructure for cross-border participation creates uncertainty by stakeholders. Finally, divergences between the main regulations applicable to research were perceived as a challenge for conducting pan-European trials and thus prevent investigational sites opening closer to the patients.

Finally, interviewees provided information on various other obstacles, such as the lack of harmonized ethical oversight in Europe, the vulnerable condition of these mostly severely sick patients that further impacts decision-making and dealing with relocation, cultural barriers, and numerous political constraints (such as UK's withdrawal from the EU (Brexit), or the inequalities between healthcare systems in Europe).

### Responsibility for Logistics and Cost Coverage

As identified above (see Challenges to participate in cross-border clinical trials), study participants saw the logistical and financial burden as the main challenge for patients seeking to participate in clinical trials abroad. Both survey and interviews investigated opinions about how best to address these hurdles.

#### Logistics

**Figure 7:**
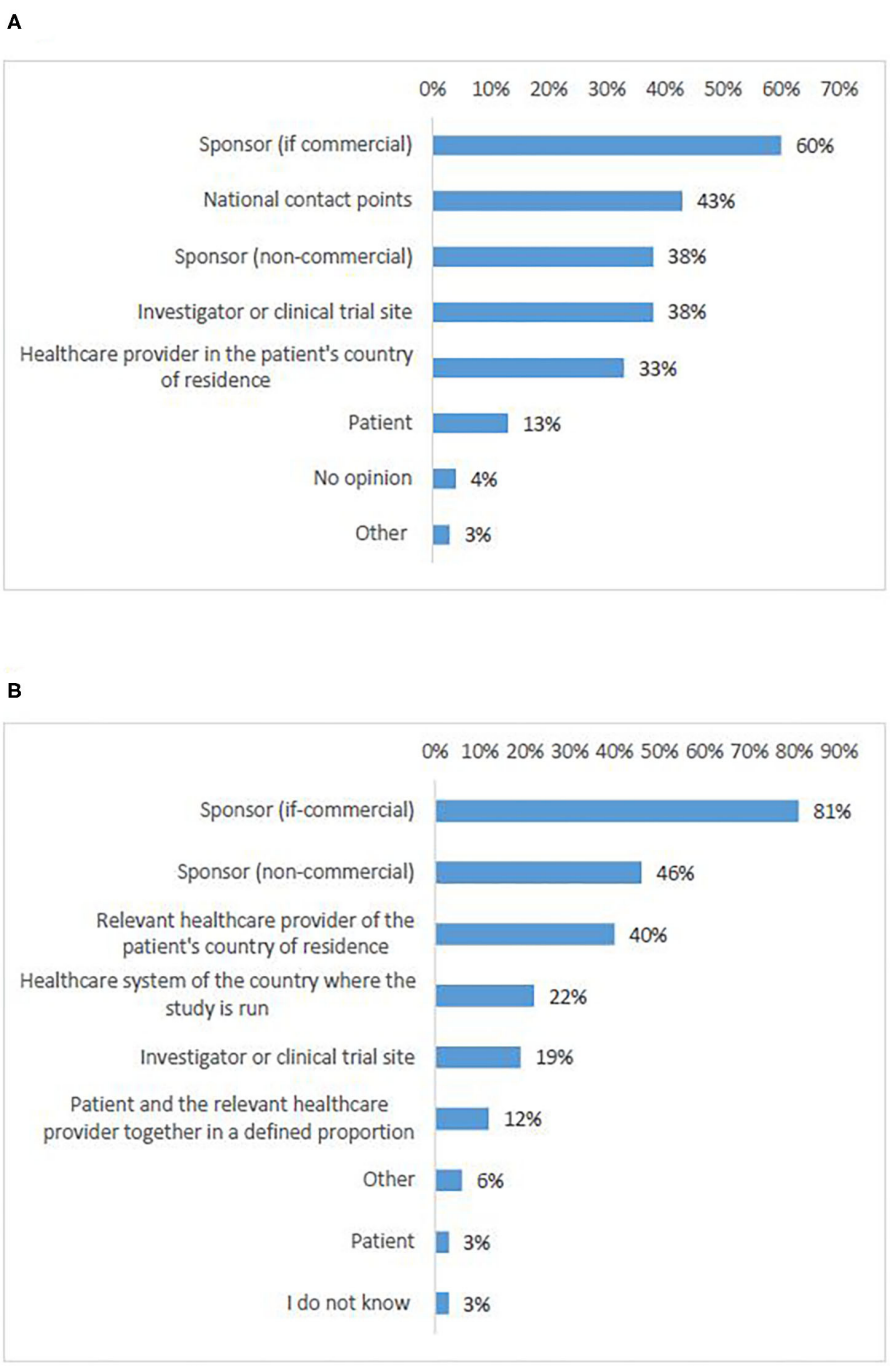
Survey respondents' opinions about who should be responsible for logistics **(A)** and cost coverage **(B)** of cross-border access to clinical trials.

According to most survey participants (55%), responsibility for logistics should be allocated to the commercial sponsor (Figure 7A, Q23). A significant number of responses proposed the NCPs (43%). Non-commercial sponsors came in third place (38%) together with investigators/clinical trial sites (38%). There was an agreement that the burden of organizing logistics should not be allocated to the patients, which was also supported by all interviewees.

“*We need to put the patients' interest at the center.” (C)*

The interviewees proposed several solution frameworks for managing logistical responsibilities (Table 3A). The ideas remained high-level, as participants acknowledged the complexity of the question and the need for further investigation of the topic at national level. While the responsibility of the sponsor (both commercial and academic) was discussed, other proposals included variations of joint support provided by home and/or host country, and aid facilitated via a new EU organization. A role for the ERNs was envisaged as well, more specifically as a way to organize patient referral. Some interviewees proposed to involve NCPs as well, however, most stated that NCPs are currently not motivated to take over a role in cross-border clinical trials.

“*[They are] resistant to this, because they say it's not their job” (B)*

**Table 3:** Interviewees' suggestions about high-level frameworks for allocation of responsibility for logistics **(A)**, and cost coverage **(B)** in cross-border access to clinical trials.

**(A) Logistics**			
**Joint model**	**Home country only**	**Sponsor only**	**EU**
(a) Home and host country. Example: the home country could organize a system for instructing the patients on practical matters related to joining a clinical trial abroad, while the host country could organize accommodation for foreign patients.	(a) Via a “foreign office” in treating hospitals that would organize travel, accommodation, and insurance matter		Via a specifically designated EU organization, or through other appropriate means.
(b) Home and host country, in collaboration with the ERNs (for patient referral).	(b) Via a “special navigator” appointed by the treating hospital to guide each patient seeking to participate in a clinical trial abroad.		
(c) Home country, supported by other interested stakeholders. Example: patient organizations	(c) Via the NCPs.		
**(B) Cost coverage**			
**Sponsor only (regardless commercial or academic)**	**Different responsibility for commercial and academic sponsors**		**Joint model (regardless whether the sponsor is commercial or academic)**
	(a) Commercial sponsor: to cover all additional costs		(a) Support by the home country.
	(b) Academic sponsor: to be supported by the patient's home country. Example: each country could allocate a specific budget for the enabling of cross-border access to clinical trials.		(b) Support by a special EU fund. Example: One interviewee (F) suggested how to collect resources for this fund–either by re-distributing money from EU's research funds, or by imposing a fee to commercial sponsors for this purpose.
			(c) Support by the host country.

The representative of a NCP who agreed to be interviewed, shared that patients sometimes seek information from them in relation to clinical trials, specifically with a focus on advanced therapy medicinal products (ATMPs). However, according to this interviewee, the responsibility for organizing logistical support should be allocated to the sponsor of the clinical trial.

#### Costs Coverage

According to most survey participants (81%), costs coverage should be the responsibility of the commercial sponsor (Figure 7B, Q24). Academic sponsors were ranked at second place with significantly fewer responses (46%). Third came the healthcare provider of the patient's home country (40%). Survey respondents agreed that the burden should not be allocated to the patients, which was supported by all interviewees.

Interviewees proposed again different framework options (Table 3B). While sponsor responsibility was largely supported, some participants expressed a fear that this may be seen as an unethical incentive for participation. Participants also suggested the creation of a special EU fund to cover expenses related to cross-border access to clinical trials. Support from the home country was also discussed, but interviewees acknowledged that not all EU Member States would have the means to do so:

“*I know the Eastern European reality, the health system here will never be able to pay for costs of sending patients abroad for a clinical trial. (…) because of the economic differences within Europe, it is not going to be possible”* (B)

### Cross-Border Access to Clinical Trials: Needed, or Not?

**Figure 8:**
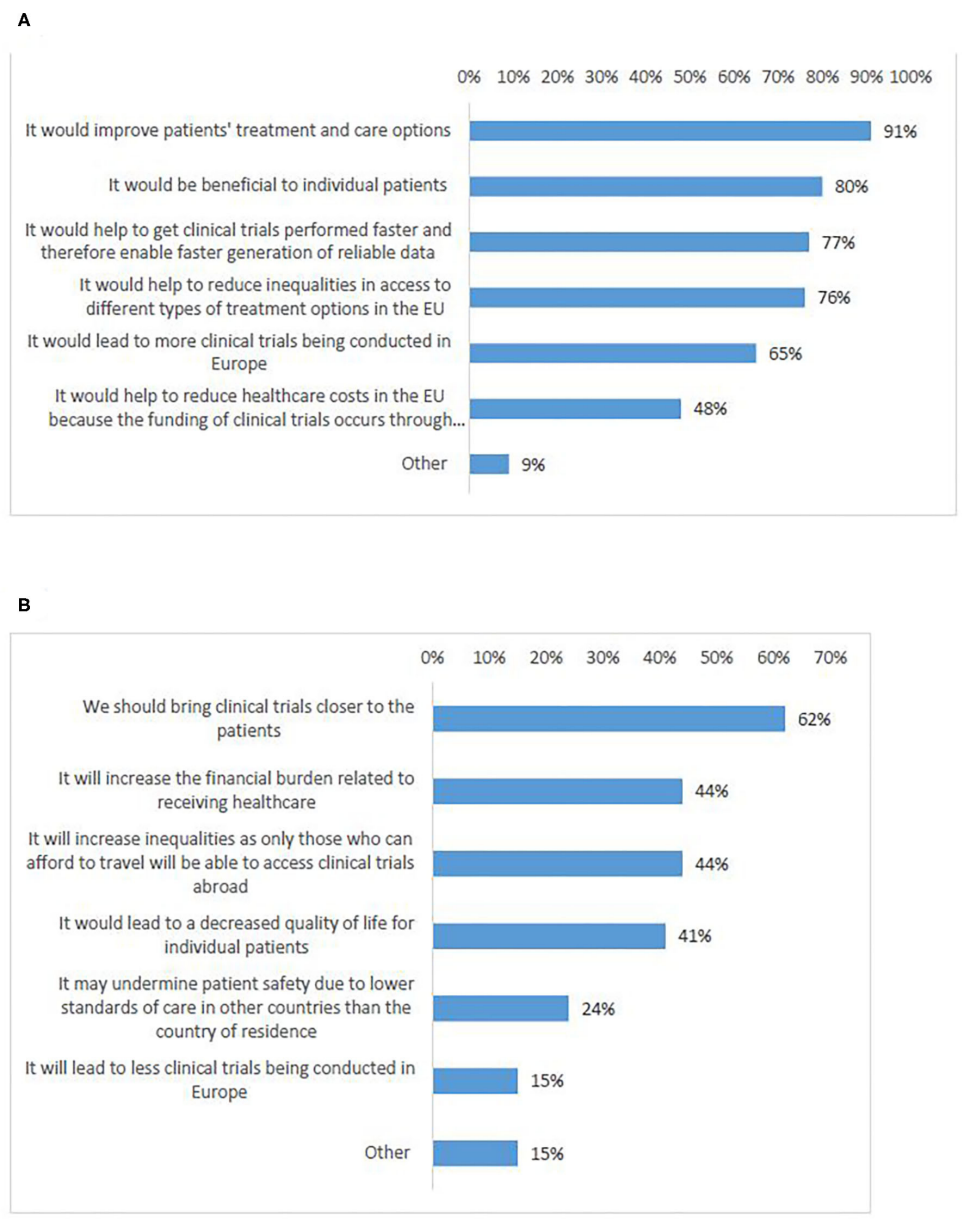
Survey respondents' views on reasons why cross-border access to clinical trials in Europe is: needed **(A)**, not needed **(B)**.

In total, 92% of survey participants agreed that cross-border participation in Europe is needed (Q19). Participants who agreed, expressed that cross-border access to clinical trials would improve European patients' treatment and care options (91%) (Figures 8A,B, Q20–21). Most of the respondents who disagreed (62%), were in favor of bringing clinical trials closer to the patients instead of having patients to travel in order to participate in clinical trials (Figure 8B). This was supported by the majority of interviewees, who partly emphasized the particular need in case of rare diseases.

“*It is needed because not all clinical trials can be open everywhere, in particular for rare diseases. So, there is a need for Member States to collaborate and for the citizens to have access to everything that is visible in the European Union. This is exactly for me the meaning of the European Union: to be together, to help ourselves, and to work together” (A)*

Several participants (A, B, E, D) specifically stated that the main aim of policy actions should be to bring the clinical trials closer to patients, either by facilitating opening sites in more countries, or by adapting the legislation for performance of remote or decentralized clinical trials.

“*When the framework is more harmonized for clinical studies, crossing borders only happens if patients are really desperate to take the extra load and I don't think the numbers are so high” (B)*

However, the majority stressed a need to find a suitable equilibrium for action that would include both facilitating cross-border access to clinical trials, on the one hand, and opening sites in more EU Member States, on the other hand.

“*I think that both increased multi-center trials are needed, and also an increased exchange of patients between the different countries.” (D)*

One interviewee (F) raised caution about the possibility that if cross-border access to clinical trials were regulated and facilitated, trials could be “*wrongly diverted.”*

“*It is very easy to say our clinical trial is only open in these three huge centers (…) there is also a kind of risk that some centers will almost make their business out of it, because of course their income is somehow related to the number of patients”*

### Should Cross-Border Access to Clinical Trials Be Limited

**Figure 9:**
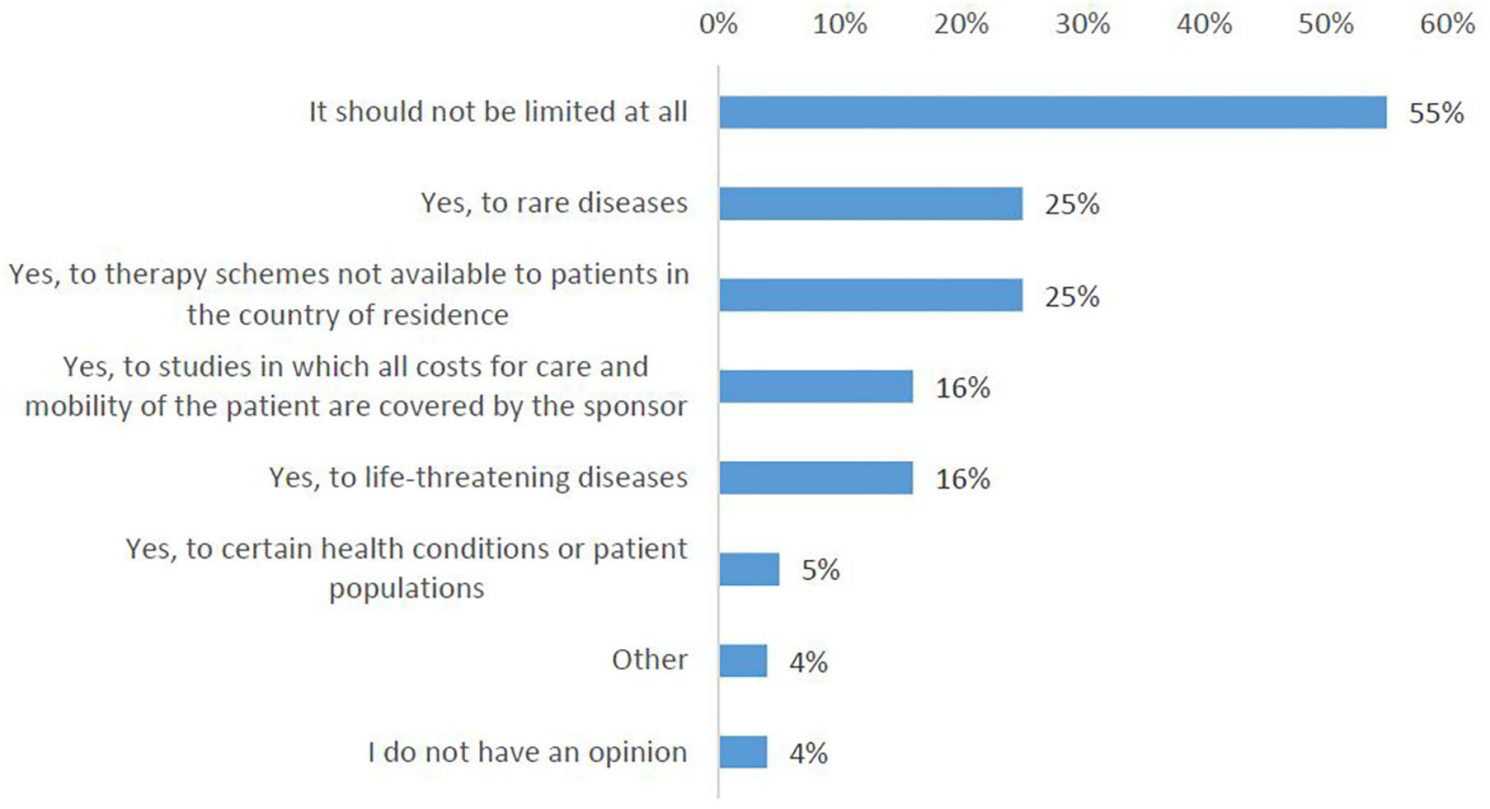
Survey respondents' opinions about limiting cross-border access to clinical trials.

More than half of the survey participants indicated that no limitations should exist (Figure 9, Q22). However, a significant number of respondents (combined) indicated that cross-border participation should be available only for rare diseases (25%) or to therapy schemes that are not available to patients in their country of residence (25%).

In the interviews, patient representatives primarily supported the idea that cross-border access to clinical trials should not be limited in any way, while investigators/physicians were generally in favor of certain limitations. They argued that the opportunity for cross-border participation should be regulated and facilitated for (1) early phase clinical trials, (2) rare diseases, and (3) precision medicine clinical trials.

“*It is only for quite specific subgroups that a cross-border invitation will be a benefit for the patient” (A)*

Other proposed limitations included facilitation on a case-by-case basis, whereby an expert opinion by a group of physicians would have to be provided; facilitation for neighboring countries based on bilateral agreements; and not allowing cross-border access in cases where an investigational site of the same trial is open in the home country of the patient.

Two of the interviewees (B, E) were of the opinion that there is no need for externally imposed limitations, as the cross-border access to clinical trials would go through a kind of “self-regulation.”

“*I somehow doubt that people would go to that length for something that is not really important. So, I would believe that there is a self-regulation in place: you only do it when you're desperate”* (B)

### Facilitation of Cross-Border Participation in Clinical Trials

#### Existing Initiatives

The interviews provided a view into the existing frameworks that facilitate cross-border access to clinical trials. The majority of examples were from the Nordic countries, e.g., the Nordic NECT (see Introduction). In Denmark and Norway, multidisciplinary national expert boards were reported to provide a new assessment of treatment options for patients who have exhausted all standard of care options. Interviewees shared that these boards assess whether there are relevant clinical trials for the patient in their home country or abroad. Another Nordic initiative concerns the development of a joint Nordic electronic information portal on Ethics Committee approvals, and is part of the three-year priority project “Nordic research collaboration for better health” (24). Some participants shared that Denmark interprets the EU social security legislation in a way that allows patients to join clinical trials abroad by using the S2 form^6^ for reimbursement of costs.

Interviewees also reported the existence of bi-lateral agreements for collaboration between University hospitals, specifically in the case of neighboring countries, such as Germany and the Netherlands.

With respect to adequate provision of information about ongoing trials (see Challenges to participate in cross-border clinical trials), the platform “FindMeCure” was given as an example. It allows patients to discover available treatment options in research, and connects them with the responsible medical team to discuss study details (25).

#### Proposals for Future Actions

**Figure 10:**
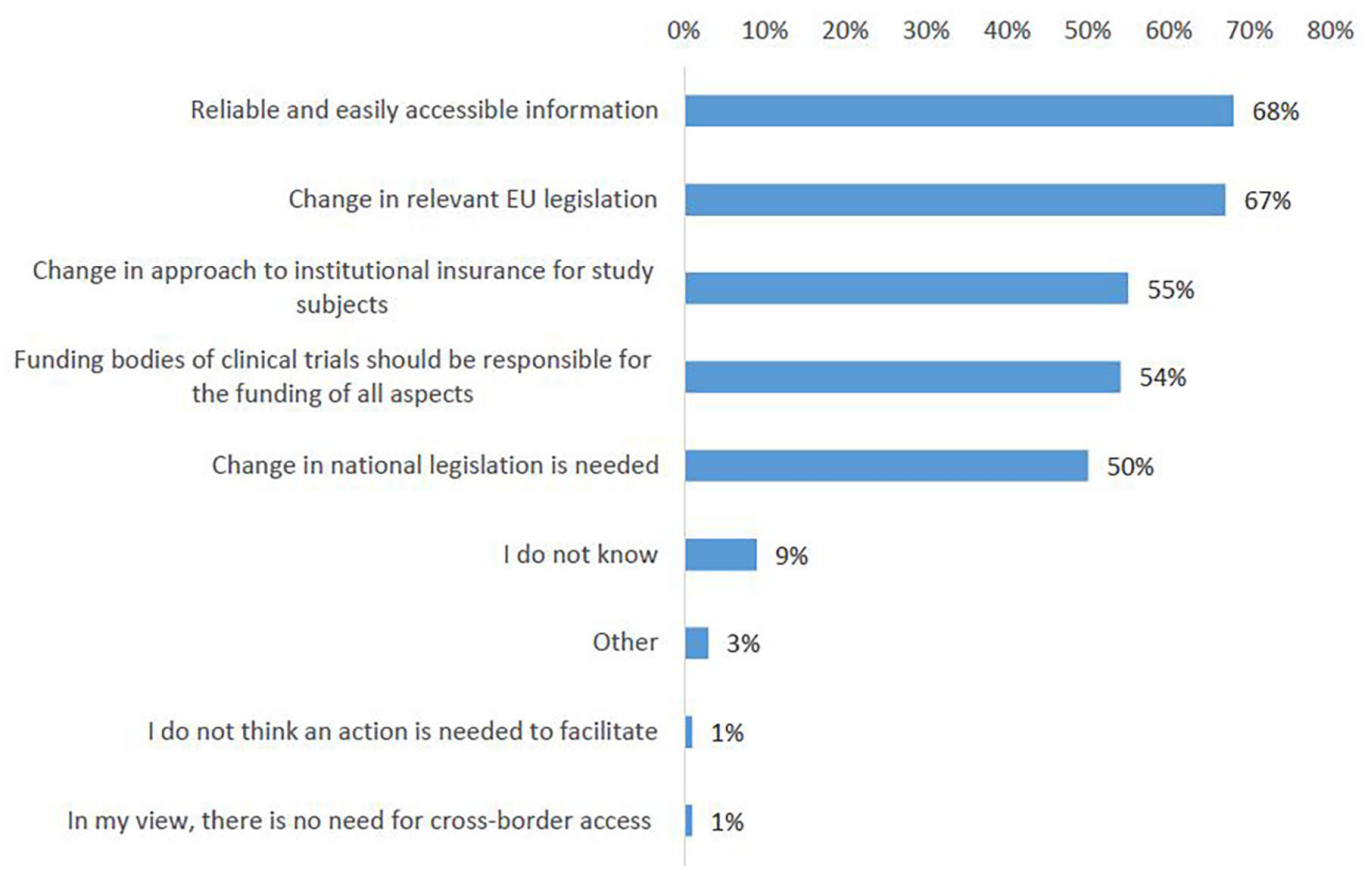
Actions that could facilitate cross-border participation in clinical trials according to survey respondents.

On the question which actions would mostly impact easier access to clinical trials abroad, the survey participants ranked highest the need for reliable and easily accessible information about the legal and administrative framework for patients crossing borders for clinical trials (68%, Figure 10, Q25). A strong support was shown for introducing a change in relevant EU legislation, in order to harmonize the conditions for cross-border access to clinical trials (67%). Almost no survey respondent indicated no need for action (Figure 10).

Interviewees suggested multiple proposals for future actions, summarized in Table 4. A few of the suggestions indirectly addressed cross-border access to clinical trials by proposing measures that would alleviate the need to travel in order to access experimental treatment. For instance, by regulating the performance of remote/decentralized clinical trials, or harmonization of the EU framework for clinical trials, which would allow the opening of more pan-EU clinical trials.

**Table 4:** Interviewees' proposals for actions that could facilitate cross-border participation in clinical trials.

**Suggestions that directly address cross-border access to clinical trials**	
Multi-stakeholder and multi-national recommendations with pragmatic solutions	•Should ideally have the status of official guidance issued or at least endorsed by an EU body, such as the European Commission or the European Medicines Agency (EMA). •Guidance development should involve all stakeholders, with patient organizations the preferred lead.
European Commission clarifying note on the Cross-border Healthcare Directive	•To include clinical research in the scope of the Directive.
Mixed model: issuing of multi-stakeholder recommendations with pragmatic solutions coupled with a change in the legislation applicable to clinical trials	•Interviewees did not provide any concrete examples as to how the legislation should be changed.
Setting up discussions between key stakeholders	As a first step toward the creation of the multi-stakeholder recommendations. Three types of discussions were proposed: 1. Discussions to clarify the role and allocation of responsibilities of different stakeholders involved in cross-border participation in clinical trials; 2. Discussions between sponsors and sites to actively foresee the feasibility for recruiting foreign patients; 3. Discussions exclusively related to a future political uptake of the issue.
Optimization of the ways relevant information is disseminated	•Establishing more optimal and user-friendly methods to inform patients about ongoing clinical trials. •National authorities to provide more and better-structured information about clinical trials. •Establishing conditions for higher awareness for treating physicians with respect to enrolling clinical trials. •More optimal use of digital services on EU and national level.
Creating a pan-European multidisciplinary tumor board or national tumor boards/expert panels	To provide a new assessment of treatment options for patients who have exhausted all available standard of care options, and to refer to suitable clinical trials at home, or abroad.
Establishing a stronger role for the European Reference Networks	
Negotiating bi-lateral agreements between neighboring countries defining the conditions for cross-border participation in clinical trials	
Utilizing the existing system of National contact points	•For provision of logistical and informational support for cross-border participation in clinical trials;
Establishing an EU fund	•To support financially cross-border participation in clinical trials.
Establishing an EU organization	•To provide support on EU level for cross-border participation in clinical trials.
Encouraging local treating physicians to refer patients to clinical trials	No concrete suggestions were provided on how this should happen in practice.
Establishing and regulating a stronger role for patient organizations	•With respect to: •provision of information •provision of logistical support •political uptake of the issue
Encouraging Member States to follow the example of Denmark and allow the use of the S2 form for clinical trials	
Local pre-screening of patients for inclusion in a trial	Making the opportunity for it available.
**Suggestions that indirectly address cross-border access to clinical trials**	
Regulating and supporting the performance of remote/decentralized clinical trials	
Harmonization of the EU framework for clinical trials, which would allow the opening of more pan-EU clinical trials	
Creation of a common ethical approval framework in the EU	

The majority of the proposals directly addressed the topic at hand. Participants generally put the highest emphasis on the need to optimize the provision of information, in particular, by the development of multi-stakeholder recommendations with pragmatic solutions. They also supported a stronger role for the European Reference Networks (see also Logistics). Changes of legislation were also discussed (e.g., by amending the scope of Directive 2011/24/EU). However, the majority of interviewees did not have legal and regulatory expertise, and acknowledged that amending the law is a long-term solution that requires further investigation and policy considerations.

## Discussion

This study showed that key stakeholders involved in clinical trials have interest in cross-border access to clinical trials. The project achieved a high response rate (396 survey respondents and 38 interviewees across the key stakeholder groups of investigators/physicians, patient representatives, pharmaceutical industry representatives, policy experts, ethics committee representatives, academic clinical trials sponsors, national contact points). All four European sub-regions were represented, although in different proportion. Investigators/physicians and patient representatives participated the most.

### Cross-Border Access to Clinical Trials Occurs Rarely but Is Needed

According to study participants, cross-border access to clinical trials currently occurs, however rarely. There are no studies or databases to cross-check this finding on an EU level. EU Member States do not collect statistical data about patients who seek participation in a trial abroad, neither in the home, nor host country. Regarding frequency, it is useful to compare our findings with information about standard patient mobility. The European Court of Auditors (ECA) confirmed that few citizens seek cross-border healthcare under the framework of Directive 2011/24/EU (~200,000 claims a year—fewer than 0.05% of EU citizens) (26).

Most participants from all stakeholder groups considered cross-border access as needed. They regarded the current system as sub-optimal and voiced an urgency to improve it. The strongest motivation for cross-border access to clinical trials was access to treatment not available in the home country of the patient. The notion “access to treatment” requires further exploration as it lacks a commonly accepted definition. In our view, access to innovative therapies should not be confused with access to healthcare in general. Gelinas et al. showed that even approved therapies may have unsurmountable obstacles to being accessible by everyone (27), making participation in a clinical trial the only option. Furthermore, they concluded that relocation of patients to a distant site would be advisable only when there is an unmet medical need. It must be noted that Gelinas et al. investigated relocation of patients for rare and ultra-rare diseases and supported a case-by-case evaluation to assess whether or not joining a trial abroad is a viable option. However, patient advocates stress the importance of participation in clinical trials in general for any indication (28).

Investigators and sponsors often listed cross-border patient participation as an option for enhancing patient recruitment. Evidence showed that as much as 80–86% of clinical trials do not reach recruitment targets (29) for a multitude of reasons (30, 31). Recruitment is a typical challenge for clinical research and not limited to a rare disease setting (32). Muts supported that in order to enhance recruitment, sponsors may consider enrolling foreign patients. However, he suggested a list of factors to consider: (1) language barriers, (2) reimbursement of travel expenses and the risk of being accused of undue incentive, (3) travel aspects that may impact patients' underlying condition, (4) the extensive administrative burden of international relocation (32). Other authors also stressed the risk that relocation could impact the study outcome measures by contributing to different responses between patients who traveled to join the trial vs. local patients (27).

### Bringing the Clinical Trial to the Patient

Most interviewees agreed that patients ideally should not have to travel in order to access experimental treatment. Bringing clinical trials closer to the patient through a simplification of the regulatory framework for clinical trials, especially for the so called remote or decentralized clinical trials were considered relevant. Literature also supports this: “*Sponsors should first consider bringing the trial to the patient by enabling remote participation, opening additional sites, or, when there exists sufficient clinical evidence, enabling expanded access programs. If insufficient infrastructure or lack of available expertise make these approaches infeasible, relocation may be considered a workable option”* (27). Denburg et al. hypothesized that opening more clinical trial sites in low- and middle-income countries may function as a lever to improve the quality of their healthcare systems (33), and Lang and Siribaddana shared a similar opinion (34). However, the decision on how many sites to have and in which location depends on a variety of factors and is more complex as it appears. For instance, early phase clinical trials, by nature, can only be conducted in specialized sites with the respective infrastructure, resources and experience. Moreover, the framework for multi-regional clinical trials is not harmonized, presenting legal, and regulatory burdens for pan-European research (24, 25). Simplifying the EU clinical trials framework is part of the solution (7). Concerning remote clinical trials, no framework exists at EU level. Both the European Medicines Agency (EMA) and the US Food and Drug Administration (FDA) recently showed support for future enabling (35, 36), but numerous complex questions must be solved before decentralized trials become a viable option.

### Countries' Attractivity for Foreign Trial Participants

Participants suggested that Western European countries are most attractive for patients seeking to participate in a clinical trial abroad. The countries of origin of patients who seek access abroad were primarily in Southern and Eastern Europe, where the healthcare systems were perceived as inferior and where, according to interviewees, fewer clinical trials are conducted. A closer look at the data shows that it is hard to make a conclusive statement about patients' flows. For instance, countries such as Germany and the United Kingdom were in the top ten both for patients seeking participation in trials abroad and for attracting foreign research subjects. Studies on patient mobility outside of a clinical trial confirm that it is difficult to draw general conclusions on its direction: “*Patient flows do not e.g., just go from South to North”* (37). Further, Glinos and Baeten identified two types of patients receiving foreign care, namely patients receiving foreign care because *they happen to be abroad when they fall ill (tourists and long-term residents) and people going abroad to seek health care, either because they live in border-regions or because of some relative disadvantage in the national health care system* (37). Inequalities between healthcare systems do exist (38). However, solving this inequality should not be confused with enabling cross-border access to clinical trials, as this involves different frameworks, needs and competences.

### Relieving Patients From Financial Burdens When Participating in Cross-Border Clinical Trials

The majority of challenges for cross-border access identified in the study are the same as the general barriers to clinical research participation within the patient's home country (39). They are also similar to hurdles for standard patient mobility (37, 40–42). The biggest challenges for cross-border participation in clinical trials were financial, administrative and legal burden, as well as lack of access to reliable and easily accessible information.

All participants agreed that the patient should not carry the financial burden. This is supported in literature (15) and recommended by the Council for International Organizations of Medical Sciences (CIOMS) guidelines (Guideline 13). Although there was support in the interviews for a primary coverage of the costs by the sponsor, especially academic sponsors and investigators/physicians stressed the financial and logistical limitations of publicly funded studies and suggested different patterns for overcoming these hurdles. Based on the ranking of results, the survey also acknowledged the limited funding abilities of non-commercial sponsors, in line with delineations by Ravinetto et al. (43). Some participants expressed a fear that sponsor reimbursement, e.g., of travel costs, may even be seen as incentive for participation. And the potential of payment unduly inducing informed consent is considered a risk (44, 45). However, the EU Clinical Trials Regulation explicitly postulates that compensation for participation is only permitted as long as it is just covering expenses and loss of earnings directly related to the participation in the clinical trial, especially relevant to consider in case of vulnerable populations.^7^ The same principle is valid in the Clinical Trials Directive, which is still in force.^8^

Based on experiences gained under the conditions of the Cross-Border Healthcare Directive, the reimbursement of expenses incurred abroad are not always covered, especially when the patients' home country has comparatively low healthcare costs (46, 47). It can be assumed that similar issues arise for reimbursement of general healthcare costs occurring in clinical trials abroad.

### Role of ERNs in Cross-Border Access to Clinical Trials

Participants largely supported a role for the ERNs in cross-border access. It has been acknowledged that the ERNs possess the capacity to concentrate expertise and thus present potential opportunities for collaboration in clinical trials (19). Already in 2018, the added value of ERNs for clinical research, specifically in the field of complex and rare diseases, was explored at a workshop, organized by EMA, the European Joint Action for Rare Diseases, and the European Commission's Directorate General for Health and Food Safety (48). In June 2019, the ERN Board of Member States agreed to the engagement of ERNs with the pharmaceutical industry, in particular on clinical trials and research projects (49). As there is no legal provision for this collaboration, the Board of Member States offered guidance on the matter, but did not address participation in studies abroad in particular (49). EURACAN (the ERN for adult rare solid cancers) presents a positive example. Productive collaborations for clinical trials were developed, particularly with EORTC (50). However, the European Commission has previously stated that in the case of ERN's, “*it is the medical knowledge that travels and not the patient”* (51). Therefore, further dialogue with all interested stakeholders is necessary to find the appropriate solution, and in the context of cancer research, a collaboration between the four cancer-related ERNs (EuroBloodNET, PaedCAN, EURACAN and GENTURIS) to address practical approaches to cross-border access to clinical trials may be helpful. Particularly when it comes to rare diseases, care cannot be separated from research due to the lack of understanding of the diseases.

Survey respondents saw a role for the NCPs. However, the response rate of NCPs in this study and insights gathered from the interviews, suggest that this currently may be complex to implement in practice. Moreover, literature shows that NCPs must further improve the way they fulfill their existing obligations under the Cross-Border Healthcare Directive, as the quality with which they provide information is variable (26, 40, 42).

While the survey suggested that a significant number of stakeholders would favor an amendment to legislation, the interviews offered more nuances for consideration. In particular, interviewees emphasized on the need for finding a rapid solution for cross-border access and, furthermore, on the complexity of the existing clinical trials framework which would require a careful debate.

In the research team's view, legislation amendments take too much time for patients in need for finding access to treatment now and in the near future. The adoption of the Cross-border Healthcare Directive, for instance, was marked by a plethora of difficult institutional compromises and a very lengthy legislative process−7 years from inception until transposition into national legislations (17). Similar or even longer timeframes would have to be envisaged for the development of a legislative framework on cross-border access to clinical trials. Amending the Cross-border Healthcare Directive with requirements for clinical trial participation abroad would be another option. However, an issue could be the fact that the Directive was intended to codify the CJEU jurisprudence in the field of patient mobility (see Introduction), of which clinical trials were not part. Study participants also suggested broadening of the scope of the Directive to clinical trials by a “note” issued by the European Commission. Indeed, the Directive envisages that the Commission is empowered to adopt delegated acts, pursuant to Article 11(5) and Article 12(5). However, the scope of delegation is very limited, namely the Commission can adopt only measures that would exclude specific categories of medicinal products or medical devices from the recognition of prescription.

### Need for a Multi-Stakeholder, Multi-National Recommendations on Cross-Border Clinical Trials

There was a consensus among interviewees and survey participants on the need for reliable and accessible information and advice on best practices, aspects to consider and risks associated with cross-border access to clinical trials. The creation of a repository of relevant national legislations, rules and articles could partially address this need. Furthermore, in the research team's view, it is necessary to create multi-stakeholder, multi-national recommendations, that bundle experience, best practice examples and expertise of knowledgeable stakeholders. As proposed by interviewees, such recommendations should be jointly developed by all stakeholder groups involved in clinical research, with balanced representation from patient organizations, representatives of the pharmaceutical industry and the academic research community, EU institutions, regulators, and payers.

### Future Research

The limited statistical data available on cross-border access to clinical trials and the exploratory character of this study make further, broader interdisciplinary research a pre-requisite before decisions on legislative next steps can be made.

In-depth legal research must be conducted prior to making normative recommendations. More specifically, a detailed look into the EU's competences in the field of cross-border research and reimbursement, and the eventual legal limits to a change would be a meaningful next step. Furthermore, legal comparative research on existing bi-lateral agreements for collaboration in the sphere of cross-border clinical trials and economic analysis would be essential.

Comprehensive data collection by commercial and academic sponsors, national healthcare providers and physicians associations on the occurrence and costs/conditions of cross-border access to clinical trials as well as a larger survey and more sophisticated interview techniques (such as Focus Groups or Delphi Groups) would be required to generate an exact presentation of the frequency of occurrence, size of the different stakeholders' needs and regional differences. It would also be important to investigate the reasons for Eastern European countries' lower response rate. Efforts are required in order to engage the representatives from this region, especially as the study suggested that the need for cross-border access is more acute there.

### Strengths and Limitations

The mixed methods triangulation design allowed us to acquire a broad understanding of the issues at hand within the project's limited time and budget. Evidence collected through interviews is not generalizable by nature; however, the answers of participants in the study should be perceived as a strong indication for the motivations and challenges concerning cross-border access to clinical trials. Moreover, the main reason for employing a mixed method design was the attempt to substantiate the key messages from the interviews with the survey's quantitative data. Due to the differences in stakeholder groups' representation in the two study arms, interview results cannot be used to explain survey answers conclusively. Nevertheless, the general alignment of interviewees and survey respondents' opinions could be perceived as a strong indication for the motivations and challenges concerning cross-border access to clinical trials.

The research project strived for equal representation of the EU countries and the stakeholder groups involved. The same amount of dissemination work was conducted to reach all regions represented in the study. Despite the high overall response rate, balanced representation was not fully achieved. First, the lower response rate from Eastern European countries could be explained with insights gathered from the interviews. Namely, we learned that the topic of cross-border access is even more difficult for patients from this region because of the more limited knowledge about the option of clinical trials, the bigger financial and languages hurdles they face, and because the treating physicians are less aware of clinical trials and less inclined to send their patients abroad. Further evaluation of the situation in Eastern European countries substantiated with more responses requires a second wave of research. Second, the lower response rates for specific stakeholder groups could be explained by a lack of interest to participate and/or lack of experience with the topic (e.g., as shown in the case of NCPs). Investigators and patient representatives were the most responsive stakeholder groups. The strong patient representation provided a clear indication of the patients' interest and needs, and their involvement in identifying challenges and searching for solutions from the first stages of investigation. However, the stakeholders representation rate may also reflect the survey dissemination strategy, which involved the networks of the research consortium members. The questionnaire was widely disseminated via investigator-, or patient-focused organizations.

Due to time- and logistical constraints, a single researcher performed the full analysis of the interview data. However, bias was limited as three researchers constructed the working analytical framework (see General design).

## Conclusion

This exploratory study set out to open the debate on cross-border access to clinical trials by investigating the needs, challenges and potential for facilitation. The majority of study participants agreed that cross-border access to clinical trials in the EU is needed but is rarely occurring at the moment. However, most interviewees were of the opinion that patients ideally should not have to travel in order to access experimental treatment. There was a consensus on the need for reliable and accessible information and recommendations regarding practical aspects of cross-border access. The development of multi-stakeholder, multi-national recommendations with information about existing options and best practices for cross-border access to clinical trials in the EU could be an efficient next step. The study sets the grounds for broader interdisciplinary research on the topic.

## Data Availability Statement

The datasets generated for this study cannot be made publicly available due to legal and ethical constraints. Study participants did not provide consent for the sharing of interview transcripts and survey questionnaires with parties other than the researchers. The survey data can be made available to other researchers only upon request, ensuring that the information is not used for secondary data analysis without the prior consent of the consortium partners who conducted this original study. The interview transcripts cannot be made available, to make sure that the confidentiality and privacy of the interviewees is preserved. However, we have prepared a comprehensive Final study report, which contains a very detailed presentation of all findings. Access to the Final study report is available on request. Requests for access should be directed to Teodora Lalova, teodora.lalova@kuleuven.be.

## Ethics Statement

Ethical review and approval was not required for the study on human participants in accordance with the local legislation and institutional requirements. The patients/participants provided their written informed consent to participate in this study.

## Author Contributions

Study conception and design was done by IK, IH, AN, DL, TL, CP, and JG. Data collection was conducted by IK, CP, and TL. Interpretation of data was performed by TL, IK, CP, IH, AN, and JG. TL drafted the manuscript. All authors were involved in revising the paper and approval of the submitted version.

## Conflict of Interest

JG was employed by Patvocates. The remaining authors declare that the research was conducted in the absence of any commercial or financial relationships that could be construed as a potential conflict of interest. The handling editor declared shared committee work, though no other collaboration with one of the authors IK, at the time of review.

## Footnotes

^1^Legal basis: Article 3(2) of the Treaty on European Union (TEU); Article 21 of the Treaty on the Functioning of the European Union (TFEU); Titles IV and V TFEU; Article 45 of the Charter of Fundamental Rights of the European Union.

^2^Article 12 of Directive 2011/24/EU.

^3^Article 12(2).

^4^Further interviewees were invited based on responses and recommendations made in earlier interviews. This technique was chosen as it is particularly useful for investigating sensitive topics which require the knowledge of insiders to identify study participants. See also (21).

^5^Countries were ascribed to sub-regions using the United Nations geo-scheme of Europe, created by the United Nations Statistics Divisions, available at: https://www.worldatlas.com/articles/the-four-european-regions-as-defined-by-the-united-nations-geoscheme-for-europe.html.

^6^Portable Document S2 (PD S2) on “Entitlement to scheduled treatment” certifies the entitlement to planned health treatment in a Member State other than the competent Member State of the insured person, based on the procedures determined by EU rules on the coordination of social security systems: Regulation (EC) No 883/2004 and Regulation (EC) No 987/2009.

^7^Article 31(1)(d), Article 32(1)(d) and Article 33(d) of Regulation (EU) No 536/2014 on clinical trials on medicinal products for human use.

^8^Article 4(d) and Article 5(d) of Directive 2001/20/EC.
