# Supplementary material for: Cross-Border Access to Clinical Trials in the EU: Exploratory Study on Needs and Reality
Source: Front Med (Lausanne). 2020 Oct 22;7:585722. doi: 10.3389/fmed.2020.585722 (PMC7642582; doi:10.3389/fmed.2020.585722)
Supplement: Supplementary file 1 [file Data_Sheet_1.DOCX]

|  | Eastern Europe | № | Northern Europe | № | Southern Europe | № | Western Europe | № |
| --- | --- | --- | --- | --- | --- | --- | --- | --- |
|  | Bulgaria | *670* | Denmark | *727* | Croatia | *162* | Austria | *890* |
|  | Czech Republic | *1215* | Estonia | *321* | Cyprus | *1* | Belgium | *1256* |
|  | Hungary | *1238* | Finland | *503* | Greece | *558* | France | *774* |
|  | Poland | *974* | Ireland | *301* | Italy | *1296* | Germany | *2156* |
|  | Romania | *112* | Latvia | *337* | Malta | *6* | Luxembourg | *4* |
|  | Slovakia | *632* | Lithuania | *379* | Portugal | *510* | Netherlands | *926* |
|  |  |  | Sweden | *758* | Slovenia | *123* |  |  |
|  |  |  | United Kingdom | *1647* | Spain | *1845* |  |  |
| TOTAL: |  | ***4841*** |  | ***4973*** |  | ***4501*** |  | ***6006*** |

Supplementary material 1:

Comparison of completed clinical trials (all indications) in the EU Member States for the period 01/01/2010 – 31/12/2019, with an overview of (A) Phase Three trials, (B) Phase One trials, (C) Phase One trials for rare diseases, (D) Phase Three trials for rare diseases.

Database: EU Clinical Trials Register, <https://www.clinicaltrialsregister.eu/>

Grouping of countries per sub-regions is based on the United Nations geo-scheme for Europe.

Search criteria included Age Range: Adult; Gender: Both; Trial Status: Completed

(A) Phase Three clinical trials 01/01/2010 – 31/12/2019

(B) Phase One clinical trials 01/01/2010 – 31/12/2019

|  | Eastern Europe | № | Northern Europe | № | Southern Europe | № | Western Europe | № |
| --- | --- | --- | --- | --- | --- | --- | --- | --- |
|  | Bulgaria | *17* | Denmark | *32* | Croatia | *0* | Austria | *33* |
|  | Czech Republic | *42* | Estonia | *4* | Cyprus | *0* | Belgium | *92* |
|  | Hungary | *47* | Finland | *13* | Greece | *20* | France | *89* |
|  | Poland | *35* | Ireland | *8* | Italy | *69* | Germany | *183* |
|  | Romania | *2* | Latvia | *2* | Malta | *0* | Luxembourg | *1* |
|  | Slovakia | *10* | Lithuania | *3* | Portugal | *14* | Netherlands | *80* |
|  |  |  | Sweden | *29* | Slovenia | *1* |  |  |
|  |  |  | United Kingdom | *173* | Spain | *136* |  |  |
| TOTAL: |  | ***153*** |  | ***264*** |  | ***240*** |  | ***478*** |

(C) Phase One clinical trials (rare diseases) 01/01/2010 – 31/12/2019

|  | Eastern Europe | № | Northern Europe | № | Southern Europe | № | Western Europe | № |
| --- | --- | --- | --- | --- | --- | --- | --- | --- |
|  | Bulgaria | *1* | Denmark | *7* | Croatia | *0* | Austria | *13* |
|  | Czech Republic | *10* | Estonia | *0* | Cyprus | *0* | Belgium | *20* |
|  | Hungary | *9* | Finland | *1* | Greece | *0* | France | *30* |
|  | Poland | *14* | Ireland | *4* | Italy | *25* | Germany | *54* |
|  | Romania | *0* | Latvia | *0* | Malta | *0* | Luxembourg | *0* |
|  | Slovakia | *1* | Lithuania | *2* | Portugal | *5* | Netherlands | *21* |
|  |  |  | Sweden | *7* | Slovenia | *0* |  |  |
|  |  |  | United Kingdom | *58* | Spain | *34* |  |  |
| TOTAL: |  | ***35*** |  | ***79*** |  | ***64*** |  | ***138*** |

|  | Eastern Europe | № | Northern Europe | № | Southern Europe | № | Western Europe | № |
| --- | --- | --- | --- | --- | --- | --- | --- | --- |
|  | Bulgaria | *66* | Denmark | *148* | Croatia | *20* | Austria | *199* |
|  | Czech Republic | *198* | Estonia | *16* | Cyprus | *0* | Belgium | *286* |
|  | Hungary | *160* | Finland | *71* | Greece | *114* | France | *181* |
|  | Poland | *149* | Ireland | *82* | Italy | *296* | Germany | *404* |
|  | Romania | *7* | Latvia | *19* | Malta | *0* | Luxembourg | *0* |
|  | Slovakia | *61* | Lithuania | *33* | Portugal | *90* | Netherlands | *237* |
|  |  |  | Sweden | *161* | Slovenia | *19* |  |  |
|  |  |  | United Kingdom | *385* | Spain | *380* |  |  |
| TOTAL: |  | ***641*** |  | ***915*** |  | ***919*** |  | ***1307*** |

(D) Phase Three clinical trials (rare diseases) 01/01/2010 – 31/12/2019
