# Supplementary material for: Cross-Border Access to Clinical Trials in the EU: Exploratory Study on Needs and Reality
Source: Front Med (Lausanne). 2020 Oct 22;7:585722. doi: 10.3389/fmed.2020.585722 (PMC7642582; doi:10.3389/fmed.2020.585722)
Supplement: Supplementary file 2 [file Data_Sheet_2.pdf]

Cross-Border Access to Clinical Trials

Welcome to Our Survey

This survey is part of the research project "Cross-Border Access to Clinical Trials in Europe", a joint study conducted by the European Forum for Good Clinical Practice (EFGCP), the European Organisation for Research and Treatment of Cancer (EORTC), KU Leuven and Patvocates, with the support of the European Federation of Pharmaceutical Industries and Associations (EFPIA).

The purpose of the survey is to gain insights into the relevance, occurrence, needs and challenges that different stakeholders are currently facing in the context of cross-border access to clinical trials in Europe.

The survey consists of 25 questions and it will take you approximately 15-20 minutes to complete it.

The deadline to complete this survey is June 30, 2019.

The results of the survey will be used solely for scientific purposes. They will be incorporated in a study report to be presented at the ECCO European Cancer Summit on 12-14 September 2019 in Brussels, Belgium, and may be published.

Participation in the survey is anonymous and voluntary. You can withdraw at any time, without any penalty or consequences.

In the scope of this survey, EORTC will process your data as data controller for the purposes that were presented to you.

The legal basis for processing your personal data is consent.

All research data will be stored for a period of 10 years. Your personal data may be stored for an additional period of time, if necessary, for the purposes of this study, or for further research on the topic.

For more information, please refer to EORTC privacy policy and SurveyMonkey privacy policy.

If you have any further questions regarding this study, please do not hesitate to contact Teodora Lalova, PhD researcher KU Leuven and EORTC fellow, at the following email address: teodora.lalova@eortc.org

Thank you for participating in this project!

* Do you consent with your personal data being processed as described above? You must click Yes in order to take the survey.

- ◯ Yes
- ◯ No

Cross-Border Access to Clinical Trials

Part I: General questions

* Q1 In which country are you based?

* Q2 To which stakeholder group do you belong?

- ◯ Representative of a patient organisation
- ◯ Individual patient/carer
- ◯ Investigator/physician
- ◯ Commercial or academic sponsor of clinical trials
- ◯ Ethics committee
- ◯ Regulator
- ◯ Other (please specify)

Q3 Please ONLY answer if you are a PHYSICIAN working in the oncology field: What is your medical specialty or indication area (e.g. solid tumour, haematological cancer, etc)?

* Q4 What is your experience with participation of patients in a clinical trial that is organised in a study site outside the patient's home country? *(multiple answers are possible)*

- ☐ I am/was a patient in a clinical trial organised in a site outside my home country
- ☐ I am/was involved as an investigator or a physician who included patients from another country
- ☐ I have been involved in designing or running a trial that allowed cross-border participation
- ☐ I have advised patients to participate in a clinical trial abroad
- ☐ My organisation has been informing patients from another country
- ☐ I am/was part of an ethics committee involved in the ethical review of a clinical trial in which patients from abroad participate(d)
- ☐ I have no experience
- ☐ Other (please specify)

* Q5 As far as you recall, do you know about clinical trials that *(please select all that apply)*

	Yes	No	N/A
explicitly foresee cross-border participation	○	○	○
explicitly forbid cross-border participation	○	○	○

Cross-Border Access to Clinical Trials

Part II: The current situation

* Q6 Have you observed an **increase** in

	Yes	No	I do not have an opinion/information on this
the **requests** for participation of foreign patients in clinical trials?	◯	◯	◯
the **inclusion** of foreign patients in clinical trials?	◯	◯	◯

Q7 Please ONLY answer if you are an INVESTIGATOR: What has been the highest percentage of foreign patients you have had in any of your clinical trials? *(if you are not an investigator, please continue to Question 8)*

◯ 80-100 %

◯ 60-79%

◯ 40-59%

◯ 20-39%

◯ 1-19%

◯ Less than 1%

◯ I cannot provide an estimation about this

* Q8 According to your knowledge, are there official statistics pertaining to the number of foreign patients participating in clinical trials conducted in your country?

◯ No

◯ I do not know

◯ Yes (please share below your source of information, if available, e.g. a link to a relevant website, an article title, etc)

* Q9 In your opinion, how frequently do patients from your country participate in clinical trials conducted abroad?

Not at all	Rarely	Moderately	Often	Very often
◯	◯	◯	◯	◯

Q10 If you have a concrete source of information for the answer provided to Question 9 above, please share it with us (e.g. a link to a relevant website, an article title, etc)

Q11 Please answer ONLY if you are a PATIENT or PATIENT REPRESENTATIVE: Which of the following factors would motivate you or patients from your patient organisation to pursue participation in a clinical trial conducted in another country? *(if you are not a patient or patient representative, please continue to Question 12)*

- [ ] **Study medication not marketed**: Access to a new treatment that is not marketed in my country of residence
- [ ] **Regimen or standard of care**: Access to a treatment scheme that is not available in my country.
- [ ] **Reimbursement**: Access to a new treatment, as my country of residence does not cover the costs for a similar treatment
- [ ] **Cost of treatment**: Access to a new treatment that is too expensive in my home country
- [ ] **No clinical trial site**: Access to a new treatment that is not available in a similar clinical trial in my country of residence
- [ ] **Center of excellence**: Access to a center in another country which I trust has a better quality of treatment and care than in my home country
- [ ] Other (please specify)

Q12 Please answer ONLY if you are a CLINICAL TRIAL SPONSOR: Which factors would motivate you to foresee in your clinical trial that investigators recruit patients from another country? *(if you are not a clinical trial sponsor, please continue to Question 13)*

- [ ] **Access to treatment**: Opportunity for a patient to get access to a promising investigational treatment or expensive technology (e.g. proton therapy) otherwise not accessible in his/her country
- [ ] **Access to excellence in care**: Opportunity for a patient to get access to higher standard of care than in the patient’s home country
- [ ] **Lower costs**: Opportunity for a patient to get abroad cheaper or free treatment which would be costly to the patient or the healthcare system in the patient’s country
- [ ] **Proximity to country border**: Geographical proximity of investigator site to patients living in a neighboring country
- [ ] **Rarity**: Incidence of patients with the protocol-required very specific in- and exclusion criteria is low
- [ ] **Enhancement of patient recruitment**: Support to the recruitment in a clinical trial in another country that struggles to recruit patients and that is not ongoing in the patient’s country
- [ ] Other (please specify)

Q13 Please answer ONLY if you are a PHYSICIAN: For which reasons would you advise a patient to seek participation in a clinical trial conducted in another country? *(if you are not a physician, please continue to Question 14)*

- [ ] **Access to treatment**: Opportunity for my patient to get access to a promising investigational treatment or expensive technology (e.g. proton therapy) otherwise not accessible in his/her country
- [ ] **Access to care**: Opportunity for my patient to get access to higher standard of care than in my patient's home country
- [ ] **Lower costs**: Opportunity for my patient to get abroad cheaper or free treatment which would be costly to the patient or the healthcare system in my country
- [ ] **Proximity to country border**: Geographical proximity of the investigator site to my patient's living in a neighboring country
- [ ] **Rarity**: Incidence of patients with the protocol-required very specific in- and exclusion criteria is low
- [ ] **Enhancement of patient recruitment**: Support to the recruitment in a clinical trial in another country that struggles to recruit patients and that is not ongoing in my patient's country
- [ ] Other (please specify)

* Q14 In your opinion, **in which other European countries** do **patients from your country** seek access to clinical trials?

- [ ] Albania
- [ ] Andorra
- [ ] Armenia
- [ ] Austria
- [ ] Azerbaijan
- [ ] Belarus
- [ ] Belgium
- [ ] Bosnia and Herzegovina
- [ ] Bulgaria
- [ ] Croatia
- [ ] Cyprus
- [ ] Czechia
- [ ] Denmark
- [ ] Estonia
- [ ] Finland
- [ ] France

- [ ] Georgia
- [ ] Germany
- [ ] Greece
- [ ] Hungary
- [ ] Iceland
- [ ] Ireland
- [ ] Italy
- [ ] Kazakhstan
- [ ] Kosovo
- [ ] Latvia
- [ ] Liechtenstein
- [ ] Lithuania
- [ ] Luxembourg
- [ ] Malta
- [ ] Moldova
- [ ] Monaco
- [ ] Montenegro
- [ ] Netherlands
- [ ] North Macedonia
- [ ] Norway
- [ ] Poland
- [ ] Portugal
- [ ] Romania
- [ ] Russia
- [ ] San Marino
- [ ] Serbia
- [ ] Slovakia
- [ ] Slovenia
- [ ] Spain
- [ ] Sweden
- [ ] Switzerland

- [ ] Turkey
- [ ] Ukraine
- [ ] United Kingdom (UK)
- [ ] I do not have an opinion/information on this

* Q15. In your opinion, **from** which other European countries do patients most likely seek access to clinical trials conducted **in your country**?

- [ ] Albania
- [ ] Andorra
- [ ] Armenia
- [ ] Austria
- [ ] Azerbaijan
- [ ] Belarus
- [ ] Belgium
- [ ] Bosnia and Herzegovina
- [ ] Bulgaria
- [ ] Croatia
- [ ] Cyprus
- [ ] Czechia
- [ ] Denmark
- [ ] Estonia
- [ ] Finland
- [ ] France
- [ ] Georgia
- [ ] Germany
- [ ] Greece
- [ ] Hungary
- [ ] Iceland
- [ ] Ireland
- [ ] Italy

- [ ] Kazakhstan
- [ ] Kosovo
- [ ] Latvia
- [ ] Liechtenstein
- [ ] Lithuania
- [ ] Luxembourg
- [ ] Malta
- [ ] Moldova
- [ ] Monaco
- [ ] Montenegro
- [ ] Netherlands
- [ ] North Macedonia
- [ ] Norway
- [ ] Poland
- [ ] Portugal
- [ ] Romania
- [ ] Russia
- [ ] San Marino
- [ ] Serbia
- [ ] Slovakia
- [ ] Slovenia
- [ ] Spain
- [ ] Sweden
- [ ] Switzerland
- [ ] Turkey
- [ ] Ukraine
- [ ] United Kingdom (UK)
- [ ] I do not have an opinion/information on this

Q16 If you have any publicly available source of information that supports the answers provided in Questions 14 and 15, please share it here (e.g. a link to an article, a title, a website etc.)

Q17 Please answer the following question if you have NOT tried yourself to get access to a trial in a different country or if you have NOT tried to help a patient to get access: (*otherwise, please continue to Question 18*)

In your opinion, do the following factors present a challenge for the cross-border access to clinical trials?

	Disagree	Neither agree nor disagree	Agree
The **uncertainty on a patient's eligibility** for the trial before the patient has traveled to the site for the screening visit	○	○	○
**Timing, frequency, and duration** of the study visits	○	○	○
**Distance** between the patient's home and the clinical trial site	○	○	○
The **logistical and financial burden** to the patient (e.g. for travel to the trial site for a screening visit and all other study visits and accommodation, higher cost of living in the other country, etc.)	○	○	○
**Language barriers** between patient/carer and investigator site staff	○	○	○
The **financial coverage** of the costs (e.g. of study medication, underlying baseline or concomitant medication, diagnostic procedures required to enroll in the trial or hospitalization of me/the patient when required in the trial)	○	○	○

	Disagree	Neither agree nor disagree	Agree
The **administrative, legal and time burden for the trial site to enroll** the patient in the study	○	○	○
The logistical burden and financial coverage of the **follow-on treatment and care at home**	○	○	○
**Transport of the investigational medicinal product** to the patient's country of residence	○	○	○
The **patients' healthcare system** and insurance does **not cover receiving care in the country of the clinical trial**	○	○	○
The **liability insurance of the clinical trial** does **not cover** patients from other countries	○	○	○
The patient's **lack of trust in the investigator** proposing the clinical trial	○	○	○
The patient's **lack of trust into the foreign country's healthcare system**	○	○	○

Other (please specify)

[ ]

Q18 If you have tried/managed for yourself to access a clinical trial in a different country or helped a patient to enroll in a clinical trial in a different country, what were the barriers that you encountered?

	Disagree	Neither agree nor disagree	Agree
The **uncertainty on my/the patient's eligibility for the trial** before I have/the patient has traveled to the site for the screening visit	○	○	○
**Timing, frequency, and duration** of the study visits	○	○	○

	Disagree	Neither agree nor disagree	Agree
**Distance** between my/the patient's home and the clinical trial site	○	○	○
The **logistical and financial burden** to me/the patient (e.g. for travel to the trial site for a screening visit and all other study visits and accommodation, higher cost of living in the other country, etc.)	○	○	○
**Language barriers** between me/patient/carer and investigator site staff	○	○	○
The **financial coverage** of the costs (e.g. of study medication, underlying baseline or concomitant medication, diagnostic procedures required to enroll in the trial or hospitalization of me/the patient when required in the trial)	○	○	○
The **administrative, legal and time burden for the trial site to enroll me/the patient** in the study	○	○	○
The logistical burden and financial coverage of the **follow-on treatment and care at home**	○	○	○
**Transport of the investigational medicinal product** to my/the patient's country of residence	○	○	○
My/the **patients' healthcare system** and insurance does **not cover receiving care in the country of the clinical trial**	○	○	○
The **liability insurance of the clinical trial** does **not cover** patients from other countries	○	○	○

	Disagree	Neither agree nor disagree	Agree
My/the patient’s **lack of trust in the investigator proposing the clinical trial**	◯	◯	◯
My/the patient’s **lack of trust in the foreign country’s healthcare system**	◯	◯	◯

Other (please specify)

* Q19 Please indicate what do you think about the following statement:

Cross-border participation to clinical trials in Europe is needed

(If you agree, please continue to Question 20, if you disagree, please continue to Question 21)

- ◯ I agree *(please answer Question 20)*
- ◯ I disagree *(please answer Question 21)*

Q20 If you agreed in Question 19, please select the reason(s) for which, in your view, cross-border participation in clinical trials is needed in Europe *(multiple answers are possible)*

- ☐ It would improve European patients’ treatment and care options
- ☐ It would help to reduce inequalities in access to different types of treatment options in the EU
- ☐ It would lead to more clinical trials being conducted in Europe
- ☐ It would be beneficial to individual patients (e.g. by prolonging their life, providing them with better quality of life, or curing them)
- ☐ It would help to get clinical trials performed faster and therefore enable faster generation of reliable data
- ☐ It would help to reduce healthcare costs in the EU because the funding of clinical trials occurs through other sources than routine healthcare
- ☐ Other (please specify)

Q21 If you disagreed in Question 19, please select the reason(s) for which, in your view, cross-border participation in clinical trials is not needed in Europe *(multiple answers are possible)*

- [ ] It will increase the financial burden related to receiving healthcare (e.g. due to the additional costs for travel and accommodation)
- [ ] It will increase inequalities as only those who can afford to travel will be able to access clinical trials abroad
- [ ] It will lead to less clinical trials being conducted in Europe
- [ ] It would lead to a decreased quality of life for individual patients (for reasons such as being away from their family, anxiety linked to being treated in a foreign setting, etc.)
- [ ] It may undermine patient safety due to lower standards of care in other countries than the country of residence
- [ ] We should work to bring clinical trials closer to the patients (i.e. more sites opening in more EU countries), instead of having patients travel in order to participate in clinical trials
- [ ] Other (please specify)

* Q22 In your view, should cross-border access to clinical trials be limited? *(multiple answers are possible)*

- [ ] Yes, to rare diseases
- [ ] Yes, to life-threatening diseases
- [ ] Yes, to certain health conditions or patient populations (please, provide an example in the "Other" box at the end of the list)
- [ ] Yes, to therapy schemes not available to patients in the country of residence
- [ ] Yes, to studies in which all costs for care and mobility of the patient are covered by the sponsor (commercial or non-commercial) of the study
- [ ] No, it should not be limited at all
- [ ] I do not have an opinion
- [ ] Other (please specify)

* Q23 In your view, who should organise the logistics of clinical trial participation of patients coming from another country? *(multiple answers are possible)*

- [ ] The patient
- [ ] The investigator or clinical trial site
- [ ] The sponsor (in case it is a commercial one)
- [ ] The sponsor (in case it is a non-commercial one)
- [ ] The official National Contact Point (as set up by Member States on the basis of Directive 2011/24/EU on patients' rights in cross-border healthcare)
- [ ] The relevant healthcare provider in the patient's country of residence
- [ ] I do not have an opinion
- [ ] Other (please specify)

* Q24 In your view, who should cover the costs for clinical trial participation of patients coming from another country, e.g. investigational medicinal product, baseline treatment, hospital stay, travel, follow-on care at home? *(multiple answers are possible)*

- [ ] The patient
- [ ] The investigator or clinical trial site
- [ ] The sponsor (in case it is a commercial one)
- [ ] The sponsor (in case it is a non-commercial one)
- [ ] The relevant healthcare provider of the patient's country of residence
- [ ] The healthcare system of the country where the study is run
- [ ] The patient and the relevant healthcare provider together in a defined proportion
- [ ] I do not know
- [ ] Other (please specify)

* Q25 In your view, which actions would facilitate cross-border access to clinical trials?*(multiple answers are possible)*

- [ ] A change in national legislation is needed to include the conditions for cross-border access to clinical trials
- [ ] A change in relevant EU legislation is needed in order to harmonize the conditions for cross-border access to clinical trials within the EU.
- [ ] A change in approach to institutional insurance for study subjects to include patients from other EU countries by default
- [ ] Funding bodies (sponsoring pharmaceutical companies and public funding organisations) of clinical trials should be responsible for the funding of all aspects of patients' participation in a clinical trial across the border
- [ ] Reliable and easily accessible information for patients, physicians and patient organisations about the legal and administrative framework for patients crossing borders for clinical trials
- [ ] I do not think an action is needed to facilitate cross-border access to clinical trials
- [ ] In my view, there is no need for cross-border access to clinical trials
- [ ] I do not know
- [ ] Other (please specify)

Thank you for your participation! If you have further comments or suggestions, please share them with us below:
