# Supplementary material for: Cross-Border Access to Clinical Trials in the EU: Exploratory Study on Needs and Reality
Source: Front Med (Lausanne). 2020 Oct 22;7:585722. doi: 10.3389/fmed.2020.585722 (PMC7642582; doi:10.3389/fmed.2020.585722)
Supplement: Supplementary file 3 [file Data_Sheet_3.docx]

Interview Guide

**Introduction to Interview Guide**

- **Present yourself (name and affiliation)**
- **Thank the interviewee** for their participation in the project.
- Explain **very** **briefly the purpose** of the interview, state its duration (apprx 1 h), and where the results will be implemented.
  - *With this interview we want to* ***study the relevance, occurrence, needs and obstacles to cross-border access to clinical trials in Europe, in order to gain insights as to whether there is a need for regulatory action in this field.***
  - *The results will* ***be incorporated*** *in a study report to be presented at the the ECCO European Cancer Summit on 12-14 September 2019 in Brussels, and also in possible publications in academic journals.*
- Put the interviewee **at ease**:
  - *No wrong answers*
  - *Digitally recorded*
  - *Confidential, anonymous*
  - *Voluntary, do not have to answer anything they do not feel comfortable answering*
  - *They can stop the interview at any time, without having to give a reason*
- Ask the interviewee if they have any **questions** before the start of the interview.
- **Explain how the interview will proceed**, e.g*.:*

*We will start with some warming-up questions. Subsequently, we will focus on the research questions which are organized across two main themes. The first theme concerns the current situation in cross-border access to clinical trials. The second theme deals with the organizational framework of access to cross-border clinical trials.*

- **Ask for permission to turn on the recording function.**

**Warming-up questions**:

- Can you tell me a little bit about yourself and your background?
- What is your current role in your organization?
- What are your expectations of this interview?

**Part I. The current situation**

1. Please **describe your experience** with participation of patients in a clinical trial that is organized on a site for trial (hospital / other healthcare unit) outside the patient’s home country?

2. If you have observed an increase OR decrease in the requests for inclusion of patients residing in your country in clinical trials open abroad in the past 3 years, please provide your opinion about the **reasons for this increase/decrease**.

3. If you have observed an increase OR decrease in the requests for inclusion of foreign patients in clinical trials set in your country in the past 3 years, please provide your opinion about the reasons for this increase/decrease.

*Note for the interviewer*: *Require information about the baseline such as: How many clinical trials are now running in your country (all)? What are the areas of indication?*

4. ONLY if the interviewee is an investigator: In your opinion or experience, would increased cross-border clinical trial participation reduce patient recruitment timelines to a relevant extent? Why?

5. ONLY if the interviewee is an investigator: Please answer the following question: According to your knowledge, what is the number of foreign patients participating in clinical trials in your country?

*- If you have a concrete source of information for the answer provided, please share it with the research team (e.g. a link to an article, a title, a website etc.)*

6. In your opinion, what is the number of patients from your country who participate in clinical trials organised abroad?

*- If you have a concrete source of information for the answer provided, please share it with the research team (e.g. a link to an article, a title, a website etc.)*

*Not for interviewer: Question about motivation, different phrasing for different stakeholders:*

7a. ONLY if the interviewee is a patient or carer: What factors would **motivate you** to pursue participation in a clinical trial in another country?

7b. ONLY if the interviewee is a representative of a patient organization: What factors would **motivate you** to recommend to patients from your patient organization the participation in a clinical trial in another country?

7c. ONLY if the interviewee is a clinical trial sponsor: What factors would **motivate you** to foresee that your investigators recruit patients from abroad?

7d. ONLY if you are a physician: For which reasons would you recommend a patient to seek participation in a clinical trial conducted in another country?

*(Question 8 and 9 deal with the same topic, so take it like that forward)*

8. In your opinion, **in which other EU Member States** do patients from your country seek access to clinical trials?

9. In your opinion, what are the **reasons** that would motivate patients to seek access to clinical trials primarily in these countries?

*(Question 10 and 11 deal with the same topic, so take it like that forward)*

10. In your opinion, **what are the EU Member States from which patients are most likely to seek access** to clinical trials conducted in your country.

11. In your opinion, what are the **reasons** that would motivate patients from these countries to seek access to clinical trials set in your country?

12. In your view, **what are the challenges** **for patients from your country** going abroad to participate in clinical trials open abroad?

13. In your view, what are **the challenges for patients coming into your country** to participate in a clinical trial?

**Part II. Organizational framework of access to cross-border clinical trials**

14. Knowledge question (as suggested by Bettina Ryll) – Do you know whether cross-border access to clinical trials is part of the current EU legislation on cross-border healthcare (the Directive 2011/24/EU on patients’ rights in cross-border healthcare)?

15. For the following statement, please complete the phrase with your opinion on cross-border participation in clinical trials in Europe.

Cross-border participation in clinical trials in Europe is **needed**/**not needed** because ……

16. In your opinion, should cross-border access to clinical trials be **limited**?

*Note for the interviewer: When we speak about “limited”, we refer to disease area, specific health conditions, and others (depending on the answer of the interviewee).*

17. *Note for the interviewer*: *whatever the response (yes/no), follow-up with a* “**Why?**”- question.

18. In your opinion, who should be **organizing** the logistics of study participation of patients coming from abroad?

19. In your opinion, who should cover the **costs** for study participation of patients coming from abroad? (e.g., investigational medicinal product where not covered by the sponsor, baseline treatment, hospital stay, travel, follow-on care at home)

20. In your opinion, which actions would **facilitate**/ support cross border participation in CTs?

21. **How** do you **perceive the current national legislation** in your country of residence in terms of cross border access to clinical trials?

22. *Note for the interviewer*: *whatever the response (e.g. if they state that they perceive the legislation as an obstacle to cross-border access), follow-up with a* “**Why**?”- question.

23. When you think of current EU legislation, do you consider it presents an obstacle to cross border access to clinical trials, or it provides a basis for facilitation?

24. *Note for the interviewer: whatever the response, follow-up with a* “**Why**?”- question.

**Round-up questions (at the end of the interview)**

These were all the questions I had for you. Before we finish:

• Do you have anything you want to add or emphasise?

• Do you have any questions for me?

***Put off audio recording***

• Do you have a suggestion for another interesting interviewee?

• Would you feel comfortable being contacted again if we have any follow-up questions?

***Thank you for your participation****. If you have any other questions or comments, do not hesitate to contact me.*
