# Supplementary material for: Cross-Border Access to Clinical Trials in the EU: Exploratory Study on Needs and Reality
Source: Front Med (Lausanne). 2020 Oct 22;7:585722. doi: 10.3389/fmed.2020.585722 (PMC7642582; doi:10.3389/fmed.2020.585722)
Supplement: Supplementary file 4 [file Data_Sheet_4.docx]

| **Question №** | **Type of question** | **№ of survey respondents who provided an answer*** |
| --- | --- | --- |
| 1 | Compulsory  Single answer drop down | 396 |
| 2 | Compulsory  Single answer multiple choice  Option “Other” provided | 396 |
| 3 | Non-compulsory  Targeted only physicians  Open-ended | 73 |
| 4 | Compulsory  Multiple answer multiple choice  Option “Other” provided | 396 |
| 5A | Compulsory  Matrix question | 393 |
| 5B | Compulsory  Matrix question | 363 |
| 6A | Compulsory  Matrix question | 304 |
| 6B | Compulsory  Matrix question | 302 |
| 7 | Non-compulsory  Targeted only investigators  Single answer multiple choice | 126 |
| 8 | Compulsory  Single answer multiple choice | 304 |
| 9 | Compulsory  Likert scale | 304 |
| 10 | Non-compulsory  Open-ended | 41 |
| 11 | Non-compulsory  Targeted only patients and patient representatives  Multiple answer multiple choice  Option “Other” provided | 131 |
| 12 | Non-compulsory  Targeted only clinical trials sponsors  Multiple answer multiple choice  Option “Other” provided | 38 |
| 13 | Non-compulsory  Targeted only physicians  Multiple answer multiple choice  Option “Other” provided | 144 |
| 14 | Compulsory  Multiple answer multiple choice | 304 |
| 15 | Compulsory  Multiple answer multiple choice | 304 |
| 16 | Non-compulsory  Open-ended | 22 |
| 17 | Non-compulsory  Likert scale | \| Uncertainty on a patients eligibility for the trial \| 205 \| \| --- \| --- \| \| Timing/frequency and duration of the study visits \| 206 \| \| Distance between the patients home and the clinical trial site \| 206 \| \| The logistical and financial burden to the patient \| 209 \| \| Language barriers between patient/carer and investigator site staff \| 206 \| \| Financial coverage of the costs \| 207 \| \| The administrative/ legal and time burden for the trial site to enroll the patient in the study \| 203 \| \| The logistical burden and financial coverage ofthe follow-on treatment and care at home \| 201 \| \| Transport of the investigational medicinal product to the patients country of residence \| 199 \| \| The patients healthcare system and insurance does not cover receiving care in the country of the clinical trial \| 205 \| \| The liability insurance of the clinical trial does not cover patients from other countries \| 201 \| \| The patients lack of trust in the investigator proposing the clinical trial \| 199 \| \| The patients lack of trust into the foreign countrys healthcare system \| 199 \| \| Other \| 5 \| |
| 18 | Non-compulsory  Likert scale | \| Uncertainty on a patients eligibility for the trial \| 124 \| \| --- \| --- \| \| Timing/frequency and duration of the study visits \| 125 \| \| Distance between the patients home and the clinical trial site \| 126 \| \| The logistical and financial burden to the patient \| 129 \| \| Language barriers between patient/carer and investigator site staff \| 126 \| \| Financial coverage of the costs \| 129 \| \| The administrative/ legal and time burden for the trial site to enroll the patient in the study \| 125 \| \| The logistical burden and financial coverage of the follow-on treatment and care at home \| 120 \| \| Transport of the investigational medicinal product to the patients country of residence \| 119 \| \| The patients healthcare system and insurance does not cover receiving care in the country of the clinical trial \| 126 \| \| The liability insurance of the clinical trial does not cover patients from other countries \| 123 \| \| The patients lack of trust in the investigator proposing the clinical trial \| 119 \| \| The patients lack of trust into the foreign countrys healthcare system \| 120 \| \| Other \| 13 \| |
| 19 | Compulsory  Single answer multiple choice | 304 |
| 20 | Non-compulsory  Multiple answer multiple choice  Option “Other” provided | 278 |
| 21 | Non-compulsory  Multiple answer multiple choice  Option “Other” provided | 25 |
| 22 | Compulsory  Multiple answer multiple choice  Option “Other” provided | 304 |
| 23 | Compulsory  Multiple answer multiple choice  Option “Other” provided | 304 |
| 24 | Compulsory  Multiple answer multiple choice  Option “Other” provided | 304 |
| 25 | Compulsory  Multiple answer multiple choice  Option “Other” provided | 304 |

** Note that for Questions 17 and 18 a different number of respondents provided an answer to each of the options listed in the Likert scale. An overview of these options is presented here.*
